# Stunning Intricacies of RNA Editing Complexes RECC, RESC, and REH2C: Functional Organization, Developmental Regulation, and Evolutionary History in Kinetoplastid Protists

**DOI:** 10.1002/wrna.70037

**Published:** 2026-02-25

**Authors:** Suzanne M. McDermott, Julius Lukeš, Laurie K. Read, Reza Salavati, Achim Schnaufer, Sara L. Zimmer, Jason Carnes, Alasdair Ivens, Naghmeh Poorinmohammad, Nicholas J. Savill, Dave Speijer, Ken Stuart, Kristína Záhonová, Poorya Mirzavand Borujeni, Zihao Chen, Cody Goode, Sunil Kumar Sharma, Lars O’Hara, Jorge Cruz‐Reyes

**Affiliations:** ^1^ Seattle Children's Research Institute Seattle Washington USA; ^2^ Department of Pediatrics University of Washington School of Medicine Seattle Washington USA; ^3^ Institute of Parasitology, Biology Centre Czech Academy of Sciences České Budějovice (Budweis) Czech Republic; ^4^ Faculty of Science, University of South Bohemia České Budějovice (Budweis) Czech Republic; ^5^ Department of Microbiology and Immunology School of Medicine and Biomedical Sciences, University at Buffalo Buffalo New York USA; ^6^ Institute of Parasitology, McGill University Ste. Anne de Bellevue Quebec Canada; ^7^ Department of Biochemistry McGill University Montreal Quebec Canada; ^8^ Institute of Immunology and Infection Research, School of Biological Sciences, University of Edinburgh Edinburgh Scotland UK; ^9^ University of Minnesota Medical School, Duluth Campus Duluth Minnesota USA; ^10^ Centre for Immunity, Infection and Evolution, School of Biological Sciences, University of Edinburgh Edinburgh Scotland UK; ^11^ Medical Biochemistry, AmsterdamUMC, UvA Amsterdam the Netherlands; ^12^ Department of Global Health University of Washington Seattle Washington USA; ^13^ Division of Infectious Diseases, Department of Medicine Faculty of Medicine and Dentistry, University of Alberta Edmonton Canada; ^14^ Department of Parasitology Faculty of Science, Charles University, BIOCEV Czech Republic; ^15^ Life Science Research Centre, Department of Biology and Ecology Faculty of Science, University of Ostrava Ostrava Czech Republic; ^16^ Royal Botanic Garden Edinburgh Scotland UK; ^17^ Department of Biochemistry and Biophysics Texas A&M University College Station Texas USA

**Keywords:** developmental regulation, editing complexes, kinetoplastids, RECC, REH2C, RESC, RNA editing

## Abstract

RNA metabolism in kinetoplastid protists (Kinetoplastea), including trypanosomes and *Leishmania*, involves unique post‐transcriptional mitochondrial RNA editing that creates translatable mRNAs through uridine (U) insertions and deletions (U‐indels) directed by antisense guide RNAs (gRNAs). Like other biological processes that require specific RNA targeting, this system faces several challenges beyond coordinating its many components: assembling mRNA‐gRNA hybrids, recognizing hundreds of sites, and accurately distinguishing pre‐edited, partially edited, and fully edited transcripts in the mitochondrial environment. In parasites such as *Trypanosoma brucei*, significant energetic adaptations to different host environments also involve critical editing changes during development. The editing holoenzyme includes three molecular complexes and isoforms that carry most proteins: RNA Editing Catalytic Complexes (RECCs), which catalyze U‐indel cycles; RNA Editing Substrate Complexes (RESCs), which serve as scaffolds to coordinate the editing components; and the RNA Editing Helicase 2 Complex (REH2C), which contains key proteins involved in developmental editing regulation. However, more proteins and functions are being discovered. The editing system, best understood in *T. brucei* , shows considerable evolutionary conservation in its core machinery; however, it varies in the extent of RNA editing and the organization of mitochondrial mRNA and gRNA genes across different species. Here we explore recent progress in our understanding of RNA editing and the growing use of modern computational tools, including artificial intelligence (AI) and structural methods, to examine function, organization, developmental regulation, and evolutionary aspects of this amazing system.

This article is categorized under:

RNA Interactions with Proteins and Other Molecules > RNA‐Protein Complexes
RNA Processing > RNA Editing and Modification

## Graphical abstract

U‐indel RNA editing targets mRNA:gRNA duplexes through three key complexes that collectively govern assembly, specificity, catalysis, and developmental regulation. Modern tools, including artificial intelligence, analyze the organization, dynamics, and evolution of the remarkable holo‐editosome, opening new avenues in RNA biology and therapy.

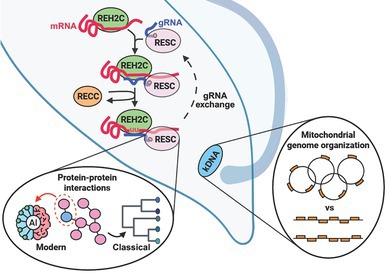

## 1 Introduction

Kinetoplastid protists (Kinetoplastea), including human‐pathogenic *Trypanosoma* and *Leishmania* species, have unique mechanisms of gene expression. Arguably, the most notable example involves extensive post‐transcriptional remodeling of the mitochondrial mRNA‐ome encoded on networks of mitochondrial, or kinetoplast, maxicircle DNA (Englund 2014). These mRNAs are modified following transcription through specific insertion and deletion of uridines (U‐indels), which generate translatable mRNAs. This distinctive process, first described in 1986 and called “RNA editing” by Rob Benne (Benne et al. 1986), has become a paradigm in RNA biology. Since then, many other RNA editing strategies have been identified in eukaryotes, especially within organelles, with deamination‐based base conversions being the most common (Tamizkar and Jantsch 2025). Initially, RNA editing seemed to challenge the central dogma of molecular genetics because the edited transcripts contained nucleotides not encoded in DNA. Pioneering studies by the Stuart, Simpson, Sollner‐Webb, and Hajduk labs in the 1990s demonstrated that kinetoplastid RNA editing is driven by proteins and guided by antisense guide RNAs (gRNAs) that base pair with the fully edited mRNA sequence through canonical and G°U pairs (Blum and Simpson 1990; Rusche et al. 1997; Sabatini et al. 1998; Seiwert et al. 1996). RNA editing is mainly studied in parasitic *Trypanosoma brucei*, which is the causative agent of Human African trypanosomiasis (HAT), where 12 of the 18 mitochondrially encoded mRNAs require editing involving hundreds of gRNA types (Cooper et al. 2019; Koslowsky et al. 2013). *T. brucei* undergoes dramatic stage‐ and substrate‐specific adaptations in its mammalian host and insect vector, including editing of specific or preferential sets of mRNA transcripts in mammalian bloodstream form and insect procyclic form life cycle stages. Such differential editing contributes to the different compositions and functions of energy‐generating systems throughout the parasite life cycle. Procyclic form parasites utilize oxidative phosphorylation for ATP generation within the insect vector and rely on editing of mRNAs that encode multiple proteins of the respiratory chain, for example, cytochromes (Gull 2001; Hendriks et al. 2000; Priest and Hajduk 1994; Schneider 2001; Vickerman 1985). In contrast, bloodstream form parasites within the mammalian host lack cytochrome activity, preferentially edit the 3′ portion of the ND7 component of respiratory complex I, predominantly if not exclusively use glycolysis for ATP production, and use the ATP synthase as an ATP hydrolysis‐driven proton pump, thereby maintaining the essential mitochondrial membrane potential (Dean et al. 2013; Schnaufer et al. 2005).

The editing holoenzyme includes numerous factors that contribute to catalysis, machinery assembly, and regulation, and most of these are currently associated with three main macromolecular complexes and isoforms. RNA Editing Substrate Complexes (RESCs), RNA Editing Catalytic Complexes (RECCs), and RNA Editing Helicase 2 Complex (REH2C) (Table 1). These complexes provide, respectively, the basic U‐specific enzymatic reactions, scaffolds for coordinating editing components, and key proteins in developmental editing regulation. Nevertheless, more proteins and functions continue to be discovered, driven by the increased power of advanced computational tools, particularly artificial intelligence (AI), and structural techniques like cryo‐electron microscopy (cryo‐EM). This comprehensive review by several experts updates earlier excellent reviews (Aphasizheva et al. 2020; Cruz‐Reyes et al. 2018; Read et al. 2016) and discusses recent breakthroughs in the current understanding of RNA editing. We thoroughly examine the function, coordination, structure, regulation, and evolutionary conservation of editing factors and genes involved in *T. brucei* and across Kinetoplastea. Overall, the RNA editing phenomenon remains surprising and brimming with intriguing questions and exciting possibilities for future discoveries. Recent research from multiple labs has enhanced our understanding of the functions and control mechanisms of trypanosome RNA editing and, more broadly, mitochondrial RNA metabolism.

**TABLE 1:** Summary of proteins in editing complexes and auxiliary factors.

Name	Alias			Function	Motifs	*TriTryp* ID
RNA editing substrate‐binding complex (RESC)						
RESC1	GRBC1	GAP2		gRNA stabilization		Tb927.7.2570
RESC2	GRBC2	GAP1		gRNA binding		Tb927.2.3800
RESC3	GRBC3	MPB8620				Tb927.11.16860
RESC4	GRBC4	MRB5390				Tb11.02.5390b
RESC5	GRBC5	MRB11870				Tb927.10.11870
RESC6	GRBC6	MRB3010				Tb927.5.3010
RESC7	GRBC7	MRB0880				Tb927.11.9140
RESC8	REMC1	MRB10130		RNA binding	ARM/HEAT	Tb927.10.10130
RESC9	REMC2	MRB1860				Tb927.2.1860
RESC10	REMC3	MRB800				Tb927.7.800
RESC11A, RESC11B	REMC4	MRB8180, MRB4150		RNA binding		Tb927.8.8180, Tb927.4.4150
RESC12	REMC5	MRB4160		RNA binding		Tb927.4.4160
RESC12A	REMC5A	MRB8170		RNA binding		Tb927.8.8170
RESC13	TbRGG2	TbRGG2		RNA binding	RGG, RRM	Tb927.10.10830
RESC14		MRB7260			PhyH	Tb927.9.7260
RESC15	PAMC1					Tb927.1.1730
RESC16	PAMC2					Tb927.6.1200
RESC17	PAMC3					Tb927.10.1730
RESC18	PAMC4					Tb927.1.3010
RESC19	MERS3	RBP7910			Z‐DNA binding	Tb927.10.7910
RNA editing catalytic complex (RECC)						
KREPA1	A1	TbMP81				Tb927.2.2470
KREPA2	A2	TbMP63				Tb927.10.8210
KREPA3	A2	TbMP42				Tb927.8.620
KREPA4	A4	TbMP24				Tb927.10.5110
KREPA5	A5	TbMP19				Tb927.8.680
KREPA6	A6	TbMP18				Tb927.10.5120
KREPB4	B4	TbMP46				Tb927.11.2990
KREPB5	B5	TbMP44				Tb927.11.940
KREPB6	B6	TbMP49				Tb927.3.3990
KREPB7	B7	TbMP47				Tb927.9.5630
KREPB8	B8	TbMP41				Tb927.8.5690
KREPB9	B9					Tb927.9.4440
KREPB10	B10					Tb927.8.5700
KREN1	N1	REN1	TbMP90			Tb927.1.1690
KREN2	N2	REN2	TbMP67			Tb927.10.5440
KREN3	N3	REN3	TbMP61			Tb927.10.5320
KRET2	T2	RET2	TbMP57			Tb927.7.1550
KREX1	X1	REX1	TbMP100			Tb927.7.1070
KREX2	X2	REX2	TbMP99			Tb927.10.3570
KREL1	L1	REL1	TbMP52			Tb927.9.4360
KREL2	L2	REL2	TbMP48			Tb927.1.3030
RNA editing helicase 2 complex (REH2C)						
KREH2	REH2			Helicase, RNA binding, regulation	DEAH‐box, dsRBDs, OB‐fold	Tb927.4.1500
KH2F1	H2F1	MRB1680		Adaptor protein	C2H2 ZnFs	Tb927.6.1680
KH2F2	H2F2				Hydratase	Tb927.6.2140
Additional proteins						
MEAT1	MEAT1			RECC‐like associated TUTase	TUTase, PAP associated	Tb927.1.1330
KPAP2	KPAP2			Putative poly(A) polymerase	NT/TUTase, PAP associated	Tb927.10.160
KREH1	REH1	mHEL61		RNA helicase	DEAD‐box	Tb927.11.8870
KMRP1	MRP1	gBP21		RNA binding		Tb927.11.1710
KMRP2	MRP2	gBP25		RNA binding		Tb927.11.13280
KRGG1	RGG1			RNA binding		Tb927.6.2230
KRBP16	RBP16			RNA binding	Cold‐shock RNA binding	Tb927.11.7900
KRBP72		MRB1590		RNA binding	ABC‐like ATPase domain	Tb927.3.1590
KRGG3	TbRGG3	MRB1820		RNA binding		Tb927.3.1820
KREAP1	REAP‐1			RNA binding		Tb927.10.9720
KRND1	RND			U‐specific 3′‐5′ exonuclease	RND, ZF‐C2H2	Tb927.9.12720
KRNP1	PRORP2			RNase P	PRORP, PPR	Tb927.11.3010
KRPN1	mRPN1			Endonuclease	RNase III	Tb927.11.8400
p22						Tb927.6.420
DRBD18				RNA binding	RRM	Tb927.11.14090

## 2 RNA Editing Catalytic Complexes

### 2.1 Compositions and Functionally Distinct Isoforms of RECCs

Soon after the initial discovery of RNA editing in *T. brucei* (Benne et al. 1986; Feagin et al. 1988), speculation for a mechanism involving a macromolecular complex containing endonucleolytic cleavage, U addition or removal, and ligation activities was proposed (Blum et al. 1990; Stuart et al. 1989). The development of in vitro editing assays (Carnes and Stuart 2007; Igo Jr. et al. 2000, 2002; Kable et al. 1996; Seiwert et al. 1996; Seiwert and Stuart 1994; Stuart et al. 1998) allowed the first detection of macromolecular complexes containing these enzymatic activities (Corell et al. 1996; Rusche et al. 1997), now termed RECCs (formerly ~20S editosomes), with biochemical purification, mass spectrometry, and bioinformatics analyses eventually revealing the identities of ~20 individual protein components (Madison‐Antenucci et al. 1998; Panigrahi, Allen, et al. 2003; Panigrahi, Gygi, et al. 2001; Panigrahi, Schnaufer, et al. 2001, 2003; Rusche et al. 1997; Stuart et al. 2004, 2002; Worthey et al. 2003) (Table 1). These proteins share sets of domains or sequence motifs (Panigrahi, Schnaufer, et al. 2003; Worthey et al. 2003), and several proteins also have predicted intrinsically disordered regions (IDRs) (Davidge et al. 2023; Park, Budiarto, et al. 2012; Wu et al. 2011). Knockdown/knockout plus site‐directed and random mutagenesis studies, combined with in vitro assays as well as poisoned primer extension, qRT‐PCR, and RNA‐seq for multiple RECC proteins have confirmed their enzymatic activities (Carnes et al. 2017, 2005, 2008; Carnes and Stuart 2007; Ernst et al. 2009, 2003; McDermott, Guo, et al. 2015; Schnaufer et al. 2001; Trotter et al. 2005; Wang et al. 2003). They also revealed essential editing functions for most proteins and their specific domains, including for those that lack apparent catalytic motifs (Babbarwal et al. 2007; Carnes et al. 2022; Carnes, Schnaufer, et al. 2012; Davidge et al. 2023; Drozdz et al. 2002; Guo et al. 2012, 2010, 2008; Huang et al. 2002; Law et al. 2005, 2007; McDermott et al. 2019; McDermott, Guo, et al. 2015; McDermott and Stuart 2017; Salavati et al. 2006; Tarun Jr. et al. 2008; Wang et al. 2003). Eight proteins contain an RNase III motif, an RNase III Associated Motif (RAM), and a U1‐like zinc finger (ZnF). These include the proteins in three heterodimeric endonucleases KREN1/KREPB8 (N1/B8), N2/B7, N3/B6, and the B4 and B5 proteins, of which only N1, N2 and N3 conserve residues that are essential for catalytic activity in other organisms and have functional catalytic domains (Carnes et al. 2022; Carnes, Schnaufer, et al. 2012; McDermott, Carnes, and Stuart 2015; McDermott et al. 2019, 2016, 2024; McDermott, Guo, et al. 2015; McDermott and Stuart 2017). RNase III domain dimers usually cleave double‐stranded RNA, thus the heterodimeric endonucleases with only one catalytic subunit may limit cleavage to just the mRNA strand in mRNA‐gRNA heteroduplexes (Nicholson 2014). Furthermore, these heterodimeric RNase III arrangements stabilize the N1‐N3 proteins (Carnes et al. 2022; McDermott et al. 2019; McDermott and Stuart 2017) and may therefore also enable non‐catalytic pseudoenzyme regulation of endonuclease activities (McDermott et al. 2019). The editing endonucleases are in three similar but functionally distinct RECCs; the dimeric endonuclease containing N1 is exclusively in RECC1, and those with N2 and N3 are exclusively present in RECC2 and RECC3, respectively. It was shown in vivo and in vitro that RECC1, which also uniquely contains the X1 exoUase, catalyzes deletion editing, while RECC2 and RECC3 both catalyze insertion editing but with differing specificities (Carnes et al. 2017, 2005, 2008; Trotter et al. 2005). Each of the three RECCs also contain 12 proteins in common that include the X2 exoUase, the T2 TUTase, and L1 and L2 RNA ligase enzymes (Aphasizhev et al. 2002; Ernst et al. 2003; Kang et al. 2005; McManus et al. 2001; Panigrahi, Gygi, et al. 2001; Schnaufer et al. 2003, 2001), in addition to several other, non‐catalytic, proteins. These non‐catalytic proteins include A1‐A6, which all have a C‐terminal OB‐fold, which is essential in A3 (Davidge et al. 2023; Guo et al. 2008), while the three largest of these also contain two C2H2 ZnF domains, which are essential in A2 and A3 (Davidge et al. 2023; Guo et al. 2010, 2008; Kang et al. 2004; Panigrahi, Schnaufer, et al. 2001; Worthey et al. 2003). Importantly, many of the non‐catalytic proteins, including B4, B5, A3, and A6, and their domains are required to maintain integrity of RECCs (Babbarwal et al. 2007; Davidge et al. 2023; Guo et al. 2008; McDermott, Guo, et al. 2015; McDermott and Stuart 2017; Salavati et al. 2006; Tarun Jr. et al. 2008; Wang et al. 2003). Overall, the three RECCs bristle with RNA and protein binding domains, including IDRs, that we believe stabilize RECC structure and enable specific coordinated molecular interactions that sequentially position mRNA editing sites at the catalysts' active sites.

### 2.2 RECC Protein Interactions and Structure

Identification of the proteins that comprise RECCs led to investigations into the structural organization of RECCs. The general organization of RECCs was determined by a combination of affinity purification, cell fractionation, yeast two‐hybrid, coimmunoprecipitation, and crosslinking mass‐spectrometry experiments (Carnes et al. 2011; McDermott et al. 2016; Panigrahi et al. 2006; Schnaufer et al. 2003, 2010). Notably, two stable heterotrimeric subcomplexes are present in all RECCs. One of these contains A1, L2, and T2 while the other contains A2, L1, and X2 (Schnaufer et al. 2003). A1 and A2 have been hypothesized to provide OB‐folds in trans to the ligases L1 and L2 for substrate binding (McDermott et al. 2016; Park, Pardon, et al. 2012; Schnaufer et al. 2003). Furthermore, the OB‐folds of other A proteins found in all RECCs appear to form an interaction network linking the heterotrimeric subcomplexes (Kala et al. 2012; McDermott et al. 2016; Park, Budiarto, et al. 2012; Park and Hol 2012; Park, Pardon, et al. 2012; Schnaufer et al. 2010; Wu et al. 2011) in addition to having predicted RNA chaperone activities (Voigt et al. 2018). B4, B5, A3, and A6 are also present in all RECCs and similarly interact with multiple proteins (Babbarwal et al. 2007; Carnes, Schnaufer, et al. 2012; McDermott, Guo, et al. 2015; McDermott et al. 2016; McDermott and Stuart 2017; Wang et al. 2003), which accounts for loss of RECC integrity upon their knockdown or knockout. Preliminary structural models and X‐ray crystallography structures were generated for these key interactors (Deng et al. 2005, 2004; McDermott et al. 2016; Park, Budiarto, et al. 2012; Park and Hol 2012; Park, Pardon, et al. 2012; Wu et al. 2011). These models have since been supplemented with AlphaFold structures (Carnes et al. 2022; Davidge et al. 2023; McDermott et al. 2024), but the overall RECC structure remains incomplete. Early cryo‐EM analyses had insufficient resolution to build complete detailed atomic models of RECCs (Golas et al. 2009; Li et al. 2009) leaving many questions unanswered. Exact protein stoichiometries are unclear, with the exception that there appears to be a single copy of each of N1, N2, or N3 per RECC (Carnes et al. 2011). However, evidence indicates that some proteins, for example, B5 and B4, may be present in multiple copies (Golas et al. 2009; McDermott et al. 2019, 2016; McDermott and Stuart 2017; Park and Hol 2012; Tinti and Ferguson 2022; Wu et al. 2011), and it is possible that their stoichiometries may be dynamic during the catalytic cycle of editing, and during editing at different sites.

### 2.3 RECC Dynamics

**FIGURE 1:**
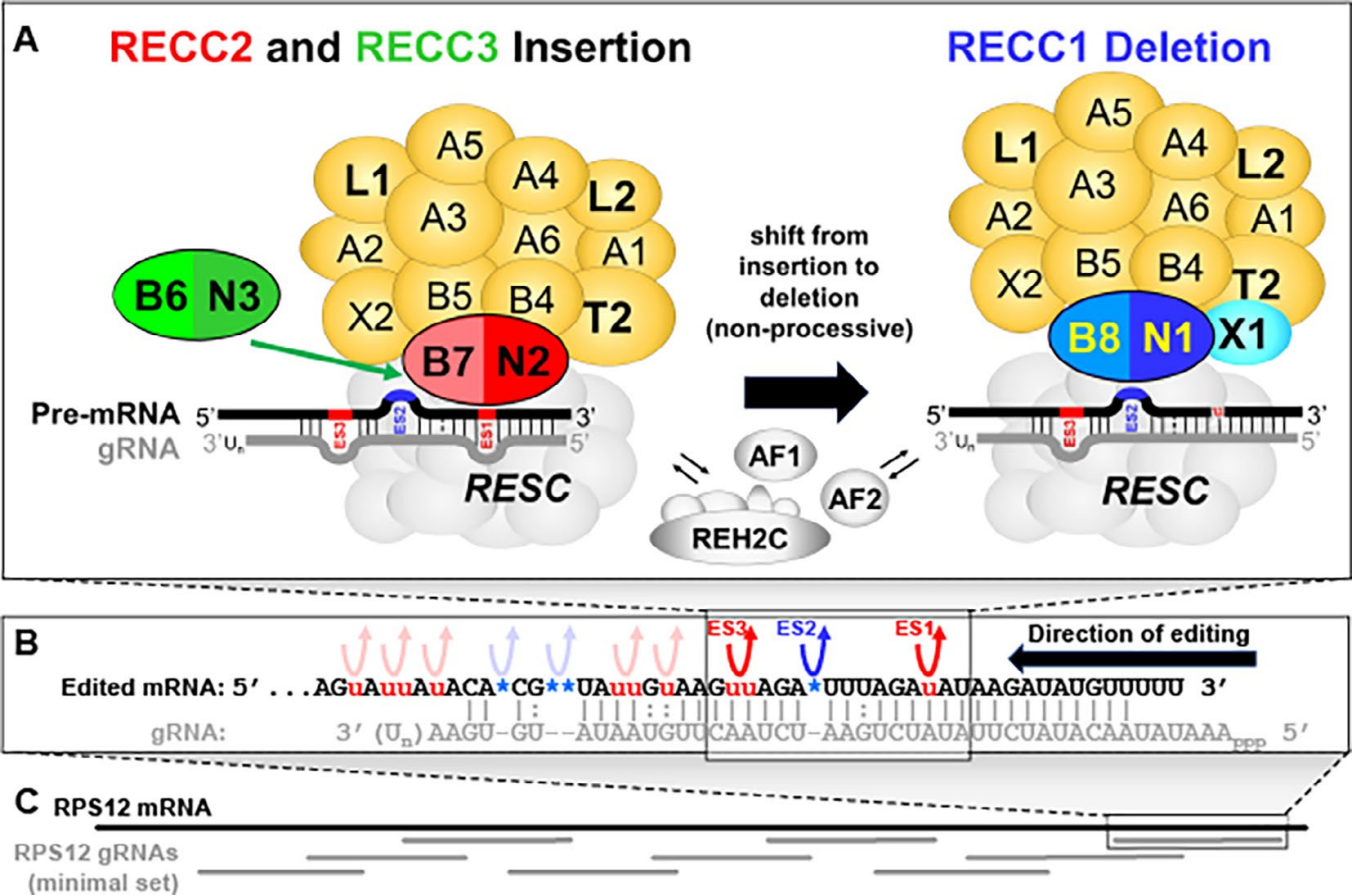
Editing complexes and overall process. (A) Diagrams (not to scale) of the 12 common RECC proteins (yellow), the N2/B7 (red) and N3/B6 (green) endonucleases that are unique to insertion RECC2 or RECC3, and the N1/B8 endonuclease and X1 exoUase (blue) that are unique to deletion RECC1. (B) Top: A single gRNA specifies editing of combinations of insertion (red) and deletion (blue) editing sites which are independently recognized and non‐processively edited by RECC2 or RECC3, and RECC1. (C) Editing of the RPS12 mRNA (black) proceeds 3′ to 5′ overall as specified by overlapping gRNAs (green). Editing of these multiple ESs in RPS12, and other edited mRNAs, requires the functions of multiprotein insertion and deletion RECCs, RESCs, REH2C, and other auxiliary factors (AFs).

Despite the characterization of the three RECCs with different endonuclease components and editing site specificities, it is unclear how they function together to edit multiple different insertion and deletion sites specified by single gRNAs in vivo. Although RECCs may be compositionally dynamic to some extent, that is, dynamically associating with auxiliary factors, they appear to be compositionally stable overall. This apparent stability of endonucleases and other common complexes and components is based on recent analyses using a modified BioID approach (Carnes et al. 2023). A consequence of this finding is that editing by different RECCs at successive insertion and deletion editing sites must be non‐processive, that is, entail successive engagement and disengagement of different RECCs rather than exchange of major components between RECC cores, such as endonuclease heterodimers (Carnes et al. 2023). In vivo dynamics also necessitate interactions with other editing complexes and auxiliary factors. Affinity purification‐based approaches have revealed that protein–protein interactions (PPIs) between RECCs and other editing complexes that interact with gRNA‐mRNA substrates including RESC and REH2C do occur, predominantly in the presence of RNA (Aphasizheva et al. 2014; Kumar et al. 2016; Madina et al. 2014; Wackowski et al. 2024). Thus, it appears that RECCs may engage and disengage at editing sites that are successively selected based on the formation of mRNA‐gRNA substrate hetero‐duplexes in the context of RESC, REH2C, and other factors (Figure 1; see also Sections 3 and 4). Additionally, once engaged, RECCs may interrogate and refine mRNA‐gRNA interactions prior to and during editing, potentially via the KREN/KREPB endonuclease U1‐like ZnFs (Carnes et al. 2022), which in the spliceosome U1C protein stabilize and fine‐tune pre‐mRNA/U1‐snRNA duplexes during splicing (Kondo et al. 2015; Muto et al. 2004; Nelissen et al. 1991).

### 2.4 RECC‐Associated Auxiliary Factors

Additional auxiliary factors have been observed to associate with RECCs and RECC proteins in various contexts, although their functional relevance is poorly understood. These include the Mitochondrial Editosome‐like complex Associated TUTase 1 (MEAT1) (Aphasizheva et al. 2009) which in *T. brucei* interacts with a subset of RECC1 proteins that do not include B8 and X1, nor insertion subcomplex components T2, A1, and L2. These MEAT1‐containing complexes can catalyze gRNA‐directed U‐insertion in vitro, albeit less efficiently than RECCs. MEAT1 was shown to be essential for both *T. brucei* bloodstream and procyclic cell viability but its knockdown via RNAi surprisingly led to moderate increases in steady‐state levels of several edited mRNAs. MEAT1 complexes also contain B10 which is orthologous to B8 (Carnes et al. 2018; Lerch et al. 2012) and has a U1‐like ZnF domain and, apparently catalytically inactive, RNase III domain. B10 and a second related ZnF/inactive RNase III protein B9 were further shown to associate with a range of RECC proteins. Specifically, B10 interacts with all RECC proteins except for B6‐B7, and B9 interacts with a similar range of proteins as MEAT1 (Lerch et al. 2012). Furthermore, these interactions are disrupted upon mutation of their RNase III domains (Carnes et al. 2018). As for MEAT1, the steady state levels of edited mRNAs are generally not affected or are moderately increased upon loss of B9 and B10, respectively (Aphasizheva et al. 2009; Carnes et al. 2018; Lerch et al. 2012). However, in contrast to MEAT1, neither B9 nor B10 is essential for *T. brucei* bloodstream or procyclic cell viability (Aphasizheva et al. 2009; Carnes et al. 2018; Lerch et al. 2012). Together these proteins and their interactions raise the possibility that MEAT1, B9, B10, and other yet unidentified transiently RECC‐associated factors might be important for the regulation of editing and/or stability of specific transcripts or even at specific editing sites. It is also possible that they have specialized essential roles in other developmental stages that have not been studied in detail, such as *T. brucei* metacyclic or stumpy forms.

### 2.5 Developmental Roles for RECCs and RECC Proteins

The phenomenon of RNA editing developmental regulation has been extensively characterized since its discovery, but the underlying mechanisms have remained elusive for decades (Feagin et al. 1988, 1987; Feagin and Stuart 1988; Koslowsky et al. 1990; Souza et al. 1993). However, key factors in stage‐specific developmental regulation have recently emerged in the separate REH2C discussed below (see Section 3) (Meehan et al. 2024, 2023). Intriguingly, published and unpublished data suggest that RECCs may also play a role in developmental regulation of editing, even though RECC protein composition appears to be essentially the same in bloodstream and procyclic cells (Carnes et al. 2011). Knockout and mutagenesis studies of multiple RECC proteins including B4‐B8 and A3 have revealed intriguing differential consequences to cell growth, RECC assembly/integrity, and editing in bloodstream versus procyclic stage cells (Carnes et al. 2022; Davidge et al. 2023; McDermott, Carnes, and Stuart 2015; McDermott et al. 2019, 2024; McDermott, Guo, et al. 2015; McDermott and Stuart 2017). Here, the growth, RECC stability, and editing phenotypes resulting from RECC protein knockout and mutagenesis are generally more severe in bloodstream than in procyclic cells, indicating that bloodstream form RECCs and RECC components are more sensitive to perturbation. This implies that bloodstream form RECC proteins and domain interactions may be more labile than in procyclics, which could potentially be related to the higher temperatures, cell doubling times, protein turnover, and different metabolic profiles required for bloodstream stage cell growth. Nevertheless, RECC protein mutations that are detrimental in procyclic but not bloodstream form cells have also been observed (McDermott, Guo, et al. 2015; McDermott et al. 2024). Taken together, these life cycle‐dependent phenotypes suggest that RECCs may be conformationally and functionally distinct between life cycle stages despite generally having the same protein compositions. This could be due to numerous stage‐specific conformations, interactions of proteins, or even post‐translational modifications within RECCs and/or between RECCs and other editing complexes, auxiliary factors, and RNA substrates. In turn, these characteristics would be expected to impact specific functions such as the rates and specificities of editing between life cycle stages. Thus, RECCs and RECC proteins may play significant roles in the developmental regulation of editing.

### 2.6 RECCs Across Kinetoplastids

Most RECC subunits are present and highly conserved in sequence and predicted structure across kinetoplastids (see Section 7). The absence of a small number of subunits, namely X2, A4, A5, B9, and B10, from some kinetoplastid species correlates with their apparently nonessential functions in *T. brucei* (Carnes, Lewis Ernst, et al. 2012; Carnes et al. 2018). However, most RECC subunits are also present in dyskinetoplastic or akinetoplastic trypanosomes that have adapted to loss of some or all of their kDNA, including *T. b. evansi* that still has functional RECCs even though they are presumably no longer required for cell survival (Carnes et al. 2015; Domingo et al. 2003; Lai et al. 2008). The presence of functional RECCs in such strains that do not contain kDNA, and therefore mitochondrial RNAs, further emphasizes that these RNAs are not required for RECC assembly. In contrast, RECC interactions with other editing complexes and factors, including RESC and REH2C, which are discussed next, are dependent on the presence of RNA (Ammerman et al. 2011; Aphasizheva et al. 2014; Hernandez et al. 2010; Madina et al. 2014).

## 3 RNA Editing Substrate Binding Complexes

### 3.1 Discovery and Definition of RESC

**FIGURE 2:**
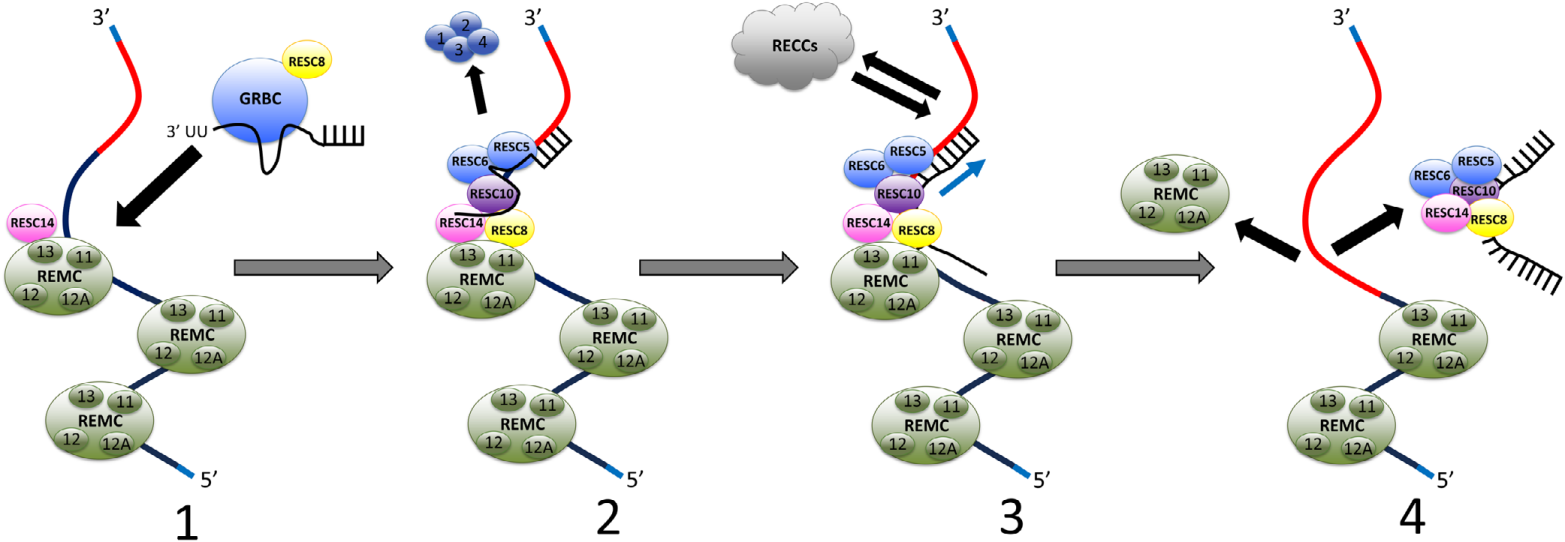
RESC is a modular and dynamic complex that undergoes numerous reorganizations during the editing cycle. RESC7 and RESC9 are omitted for simplicity. See text for details. Adapted from Wackowski et al. 2024.

RESC (formerly Mitochondrial RNA Binding Complex 1, MRB1) is a non‐catalytic complex that constitutes the platform for RNA editing and plays a key role in organizing protein–protein and protein‐RNA interactions during the editing process. RESC was identified in parallel by three groups using various pull down/mass spectrometry approaches (Hashimi et al. 2008; Panigrahi et al. 2008; Weng et al. 2008) and substantiated by numerous subsequent pulldown studies (Ammerman et al. 2011, 2013; Hashimi et al. 2009; Hernandez et al. 2010; Kafková et al. 2012). Each group identified over a dozen interacting proteins with overlapping, but varied, protein composition between groups likely due to both tagging of different proteins and differing experimental conditions. Common proteins included the homologous and interacting gRNA binding proteins, RESC1/2 (formerly GAP2/1 or GRBC1/2). RESC was first functionally linked to RNA editing by the RNAi‐mediated depletion of RESC1/2, which resulted in the loss of gRNAs and inhibition of editing of all transcripts requiring a *trans*‐acting gRNA (i.e., all but COII) (Hashimi et al. 2009; Weng et al. 2008). To better define RESC composition, a comprehensive yeast two‐hybrid analysis of all 31 putative components was performed, and the results were validated by numerous pulldowns in the presence and absence of RNase (Ammerman et al. 2012). These studies identified two distinct RESC modules, which will be referred to herein using their long‐standing names, GRBC (Guide RNA Binding Complex (Aphasizheva et al. 2014); a.k.a. MRB1 core (Ammerman et al. 2012) and RESC‐A (Liu et al. 2023) and REMC (RNA Editing Mediator Complex; Aphasizheva et al. 2014); a.k.a. TbRGG2 subcomplex (Ammerman et al. 2012) (Table 1)), along with numerous proteins envisioned as RESC organizers. The composition of GRBC and the modular nature of RESC was later validated by a comprehensive pull down/mass spectrometry study (Aphasizheva et al. 2014). The same study revealed that RESC binds gRNAs and mRNAs, while RECC does not, and showed that RECC interacts with RESC transiently and in an RNA‐dependent manner, thereby establishing RESC as the scaffold for editing (Aphasizheva et al. 2014). The vast majority of studies on RESC have been performed in *T. brucei* , where all RESC proteins, with one exception (RESC3), are essential for growth of the procyclic stage (Acestor et al. 2009; Ammerman et al. 2011, 2013; Aphasizheva et al. 2014; Dubey et al. 2021; Fisk et al. 2008; Hashimi et al. 2009; Kafková et al. 2012; McAdams et al. 2019, 2018; Weng et al. 2008). However, RESC3 is needed for optimal growth in media that forces use of oxidative phosphorylation for ATP generation (Huang et al. 2015). Where tested, RESC proteins are also essential (Ammerman et al. 2011, 2013; Fisk et al. 2008; Hashimi et al. 2009) or necessary for optimal growth (McAdams et al. 2018) in the bloodstream stage. Depletion of any RESC proteins leads to loss of editing, with distinct proteins having different effects as discussed below. RESC proteins, and apparently RESC organization, are generally conserved in kinetoplastids, such as *T. brucei, Leishmania tarentolae and Blastocrithidia nonstop* (Afonin et al. 2024; Weng et al. 2008) (see Section 7), while fewer than half of RESC proteins are identifiable in the early‐branching kinetoplastid *Perkinsela* (see Section 7). Only a few RESC proteins harbor recognizable domains: RESC13 has an RNA Recognition Motif (RRM) and RESC8 comprises canonical ARM/HEAT repeats (Fisk et al. 2008; McAdams et al. 2019; Travis et al. 2019). RESC5, RESC14, and GAP1/2 are dimethylarginine dimethylaminohydrolase, phytanoyl‐CoA dioxigenase, and RNA triphosphatase pseudoenzymes, respectively (Dolce et al. 2023; Liu et al. 2023; McAdams et al. 2018; Salinas et al. 2023). However, AlphaFold and cryo‐EM analysis revealed that numerous RESC subunits (RESC3, 4, 6, 8, 9, 10, 11A/B) have related structures comprised almost entirely of right‐handed helical structures reminiscent of HEAT repeats (Carnes et al. 2023; Liu et al. 2023). Below, we discuss the current state of knowledge of RESC organization and function, highlighting its modular and dynamic nature (Figure 2).

### 3.2 GRBC

GRBC is an RNase‐resistant particle containing six proteins (RESC1‐6). It is the RESC module that delivers gRNA to the mRNA. Central to GRBC are the RESC1 and RESC2 proteins that share 31% identity and are dependent on each other for stability (Hashimi et al. 2009). Within GRBC, RESC1/2 take care of stabilizing the gRNA population (Hashimi et al. 2009; Weng et al. 2008); the other GRBC components are dispensable as shown by the continued stability, and often increased abundance, of gRNAs upon their depletion (Ammerman et al. 2011, 2013; Aphasizheva et al. 2014; Huang et al. 2015). Specific effects of GRBC proteins on RNA editing differ slightly between studies, likely due to differing degrees of knockdown efficiency. Generally, depletion of RESC1/2 leads to a decrease in editing of all RNAs except COII (Aphasizheva et al. 2014; Hashimi et al. 2009; Weng et al. 2008). Cells with knockdowns of each of the other four GRBC components typically display widespread editing defects, often with greater effects on those RNAs requiring more gRNAs for complete editing (pan‐edited mRNAs) than those that are moderately edited (Ammerman et al. 2011, 2013; Aphasizheva et al. 2014; Huang et al. 2015). Despite requiring only a *cis*‐acting gRNA, editing of COII mRNA is decreased by 30%–60% in cells depleted of either RESC5 or RESC6, underscoring the importance of these GRBC proteins, potentially at a later step in the editing process (see below) (Ammerman et al. 2011, 2013; Aphasizheva et al. 2014). The physical independence of GRBC and RECC was revealed by several studies demonstrating that GRBC protein knockdown does not affect the integrity of RECC (Acestor et al. 2009; Ammerman et al. 2011; Huang et al. 2015; Weng et al. 2008). Further, knockdown of either RESC1/2 or RESC6 does not hinder RECC's ability to catalyze editing in vitro (Ammerman et al. 2011; Weng et al. 2008). One GRBC component, RESC3, harbors a putative transmembrane domain and is present solely in a mitochondrial membrane fraction, suggesting that RNA editing might occur in association with the mitochondrial membrane (Huang et al. 2015). Two recent cryo‐EM studies shed considerable light on the molecular basis of RESC1/2 action in gRNA binding and stabilization. Dolce et al. (2023) analyzed baculovirus purified RESC1/2 bound to in vitro synthesized gRNA, while Liu et al. (2023) purified GRBC from parasites harboring tagged RESC5. These studies showed that both RESC1 and RESC2 contain β‐barrels resembling those of RNA triphosphatases, with the inside of the tunnels possessing similarity to the triphosphate binding tunnels of mRNA capping enzymes such as *Saccharomyces cerevisiae* Cet1p. However, substitutions of two critical metal‐binding glutamic acid residues in RESC1 and RESC2 render them inactive as phosphatases. Purified RESC1/2 requires a 5′ triphosphate for RNA binding (Dolce et al. 2023), and it is this triphosphate binding activity that accounts for the discrimination of gRNA from other mitochondrial RNAs, as gRNAs are the sole 5′ triphosphorylated RNA species in *T. brucei* mitochondria (Blum and Simpson 1990; Sement et al. 2018). Cryo‐EM structures further demonstrated that RESC2 is the gRNA binding subunit of the RESC1/2 heterodimer, with the gRNA triphosphate coordinated in the RESC2 tunnel by a series of positive residues. The substitution of two of these positive residues in RESC2 likely accounts for its inability to bind gRNA. Analysis of the intact GRBC (Liu et al. 2023) (termed RESC‐A in this reference) revealed that a charged crevice is present between RESC5 and RESC6, and RESC3 and RESC4 stabilize the conformations of the RESC1/2 and RESC5/6 heterodimers. The 3′ end of the gRNA is encompassed by the RESC5/6 crevice, while the anchor and guiding regions of the gRNA form a stem similar to reported solution structures of gRNA (Hermann et al. 1997; Liu et al. 2023; Schmid et al. 1995). Overall, these data reveal how RESC1/2 stabilize gRNAs by sequestering their 5′ ends and indicate that gRNA must undergo extensive rearrangement upon association with mRNA.

### 3.3 REMC

REMC is less well defined than GRBC, as it has never been isolated and fully characterized as a discrete particle. The term refers to a group of RNA binding proteins that interact preferentially with pre‐edited mRNA: RESC11A/B, 12, 12A, and 13. Based on pulldown/mass spectrometry experiments and cryo‐EM structures of what likely constitutes the editing competent version of RESC, RESC7 and RESC9 may also be REMC constituents (Aphasizheva et al. 2014; Liu et al. 2023); however, they will not be discussed further in detail as they have not been studied sufficiently. RESC11A/B are nearly identical paralogs (referred to as RESC11 hereafter), while RESC12/12A are paralogs that differ 15% in their N‐terminus and exhibit slightly different RNA binding properties (Dixit et al. 2017; Kafková et al. 2012). REMC is defined as distinct from GRBC because RNase treatment of RESC or depletion of some RESC organizers (see below) causes these two modules to largely dissociate from one another while remaining mostly internally associated (Ammerman et al. 2012; Aphasizheva et al. 2014; McAdams et al. 2019, 2018). Moreover, knockdown of RESC13 causes partial destabilization of the RESC11 and RESC12A populations, consistent with formation of a REMC complex or complexes (Simpson et al. 2017). Distinct populations of REMCs may exist, as one study showed that RESC11 is present at significantly lower levels than RESC12A or RESC13 (Simpson et al. 2017). RESC13 has a dramatic preference for binding pre‐edited mRNA over fully edited mRNA or gRNA. Structural and molecular modeling studies of the RESC13 RRM show that this domain uses multiple binding modes to allow diffusion along U‐rich sequences, while its mode of binding to G‐rich sequences is unclear (Lemmens et al. 2025; Travis et al. 2019). Additional studies employing in vitro filter binding assays with recombinant protein also implicate the RESC13 N‐terminal domain in binding of G‐rich pre‐edited mRNA regions (Foda et al. 2012). RESC12A shows a similar preference for mRNA over gRNA in vitro, modestly favoring pre‐edited over fully edited mRNA (Kafková et al. 2012). In vivo cross‐linking studies confirmed the strong biases of RESC12, 12A, and 13 for pre‐edited mRNA, with RESC11 having a similar but less marked preference (Dixit et al. 2017; Liu et al. 2023). In keeping with their critical mRNA binding activities, RESC12A, 13, and to a lesser extent RESC11, fail to form higher‐order complexes by blue native PAGE in RNase‐treated extracts (Wackowski et al. 2024). The same study also strongly suggested the presence of multiple REMC complexes on a given mRNA based on the size of REMC‐containing complexes following GRBC dissociation. Similarly, UV cross‐linking and affinity purification (iCLAP) of RESC12 and 12A followed by high‐throughput sequencing revealed binding of these proteins in vivo along the entire lengths of six out of nine pan‐edited mRNAs, suggesting that they coat pre‐edited mRNA to mark these RNAs for editing (Dixit et al. 2017). Depletion of RESC12/12A reduces the association of RESC13 with poly(A) RNA, implicating RESC12/12A in assembly or stabilization of REMC on the mRNA (Dixit and Lukeš 2018).

The REMC proteins whose functions have been studied in any detail display both distinct and overlapping functions. Knockdown of either RESC11 or RESC13 almost exclusively and strongly hinders editing of pan‐edited mRNAs, while knockdown of RESC12/12A, which act redundantly in editing, also has modest effects on moderately edited transcripts (Acestor et al. 2009; Aphasizheva et al. 2014; Fisk et al. 2008; Kafková et al. 2012; Simpson et al. 2017). To better understand how REMC proteins modulate the editing process, the sequences of partially edited mRNAs, which constitute the majority of mRNAs in vivo, were examined. Early studies of RESC13 knockdowns revealed a dramatic defect on the 3′ to 5′ progression of editing on RPS12 mRNA and showed that editing pauses at specific sites in cells depleted of RESC13 (Ammerman et al. 2010). qRT‐PCR analysis of RESC13 knockdowns across three pan‐edited mRNAs (RPS12, A6, COIII) confirmed that RESC13 has no role in the initiation of editing, as the levels of total and pre‐edited mRNAs remained unchanged even as the corresponding fully edited mRNA decreased to 65%–90% of uninduced levels (Sortino et al. 2022). Conventional sequencing of qRT‐PCR products lacks the power to distinguish editing pauses at mRNA positions corresponding to gRNA ends, which would indicate an impact on gRNA exchange, from pauses within gRNA‐directed regions. To remedy this limitation, a dedicated bioinformatic tool termed TREAT (Trypanosome RNA Editing Alignment Tool) was developed to permit detailed analysis of high throughput sequencing data and better inform the nature of editing defects in REMC knockdowns, and thus the functions of REMC proteins (Simpson et al. 2017). Application of high throughput methods to partially edited mRNA populations allowed single nucleotide resolution of editing defects on a given mRNA as well as comparison of defects between cells depleted of either RESC11, RESC12/12A, or RESC13 (Simpson et al. 2017; Sortino et al. 2022). These highly precise analyses established that knockdown of any of these REMC factors did not cause a defect in gRNA exchange, as pausing rarely occurred at positions corresponding to gRNA ends, while the positive control RESC2 knockdown did exhibit this expected phenotype (Simpson et al. 2017; Sortino et al. 2022). Rather, progression of editing within gRNA‐directed domains was inhibited at numerous sites, now termed Exacerbated Pause Sites (EPS). Several lines of evidence highlight shared functions of RESC11 and RESC13, and distinct actions of RESC12/12A, in addition to the aforementioned impact of RESC12/12A on moderately edited mRNAs. First, EPS arising upon knockdown of RESC11 and RESC13 exhibited significant statistical overlap, while those in RESC12/12A knockdowns did not. Second, of the three REMC factors tested, only RESC12/12A depletion also led to a decrease in the initiation of RPS12 editing. Finally, editing is typically not strictly linear within a given gRNA‐directed region; a 5′ site may be edited prior to a more 3′ site within a gRNA‐directed region, and sites can likely be remodified; this phenomenon gives rise to non‐canonically edited junctions at the leading edge of editing (Ammerman et al. 2010; Carnes et al. 2023; Koslowsky et al. 1991; Simpson et al. 2017; Zimmer et al. 2018). Analysis of editing pathways in high‐throughput datasets showed that knockdown of RESC11 and RESC13, but not RESC12/12A, causes an increase in strict site‐by‐site (linear) 3′ to 5′ progression. We note that, in addition to confirming the coordinated actions of RESC11 and RESC13 in non‐linear editing, the correlation between increased linear editing and an overall decrease in editing progression strongly implies that junctions are an intrinsic and key feature of editing.

An additional role of some REMC proteins, gleaned by the analysis of high throughput sequence data, is to properly restrict the region of active editing. Upon knockdown of RESC12/12A or RESC13, numerous incidences of aberrant editing far 5′ of large pre‐edited regions in RPS12, A6, and COIII mRNAs were observed (Simpson et al. 2017; Sortino et al. 2022). In this case, RESC11 did not exhibit the same phenotype. Thus, RESC13 partners with different REMC proteins to facilitate distinct facets of 3′ to 5′ editing progression. RESC13 and RESC12/12A similarly impact progression through a gRNA‐defined region; RESC13 and RESC11 act similarly to restrict editing action to a specific region of mRNA and inhibit aberrant 5′ editing. Both of these effects almost certainly involve modulation of mRNA, gRNA, and/or gRNA‐mRNA structure. In this context, it is notable that RESC13 exhibits both RNA annealing and chaperone/melting activities, conferred by its G‐rich and RRM domains, respectively (Ammerman et al. 2010; Foda et al. 2012; Travis et al. 2019). Both activities are likely critical to RESC13 function during the editing process. Overall, REMC preferentially binds pre‐edited mRNAs apart from GRBC. REMC proteins exhibit functional heterogeneity, and physically distinct REMC complexes may exist. Together, REMC proteins ensure proper editing progression both within a given gRNA‐directed region and across an mRNA.

### 3.4 RESC Dynamics and Organizer Proteins

Numerous lines of evidence point to a highly dynamic RESC that undergoes rearrangements during the editing process. First, RNase treatment largely dissociates GRBC and REMC into distinct entities (Ammerman et al. 2012; Aphasizheva et al. 2014). In general, under these conditions, GRBC remains intact, while some intra‐REMC contacts are slightly reduced (Ammerman et al. 2012; Aphasizheva et al. 2014; Kafková et al. 2012). Second, similar results are observed when either RESC8 or RESC14 is depleted by RNAi, thus leading to the characterization of these two proteins as RESC organizers (McAdams et al. 2019, 2018). Third, GRBC was isolated from RNase‐treated extracts and subsequently characterized by cryo‐EM as a unique particle separate from the remainder of RESC (Liu et al. 2023). Together, these findings imply that GRBC and REMC continuously dissociate and associate in an orchestrated manner during editing (Figure 2). Indeed, the editing process, in which GRBC delivers gRNAs to the mRNA, appears to necessitate these dynamic interactions because a given GRBC‐bound gRNA needs to be replaced after its complete utilization, and some mRNAs take dozens of gRNAs to accomplish complete editing. To establish the roles of organizer proteins RESC8 and RESC14 in this process, blue native gel studies of differently tagged RESC complexes in the presence or absence of RESC8 or RESC14, combined with TurboID proximity analysis in the same cell lines, were employed (Wackowski et al. 2024). These studies showed that RESC14 is required for the incorporation of GRBC components RESC6 and RESC2, as well as RESC8, into the largest RNA‐containing complexes. However, RESC8 is not required for RESC14 to associate with these large complexes (Figure 2, Step 1). In the absence of RESC14, RESC6, RESC2, and RESC8 dissociate into similarly sized complexes, consistent with a RESC8‐GRBC interaction (Figure 2, Step 1). This RESC8‐GRBC interaction is likely a transient event that rapidly mediates GRBC and REMC association as RESC8 is absent from the cryo‐EM structure of GRBC (Liu et al. 2023). Finally, the absence of RESC8 destabilizes, but does not entirely preclude, RESC6 association into large complexes. TurboID data suggested continuous failed GRBC‐REMC contacts in the absence of RESC14. Together, these data support a model in which RESC14 association with REMCs on the mRNA is required for recruitment of GRBC to REMC; RESC8 interacts with GRBC to ultimately stabilize the GRBC‐REMC interaction. High throughput sequencing data further support the concerted actions of RESC8 and RESC14 in promoting GRBC‐REMC association (McAdams et al. 2019; Wackowski et al. 2024). Importantly, TurboID confirmed that RECC fails to associate with RESC if RESC is disorganized by RESC14 depletion.

The cryo‐EM studies revealed that RESC undergoes a substantial reorganization following the GRBC‐REMC interaction (Liu et al. 2023). These authors identified what they consider to be the Editing Competent form of RESC (EC‐RESC; referred to as RESC‐B in Liu et al. 2023), consisting of RESC5‐RESC14, except for RESC12/12A, which are released from EC‐RESC by RNase treatment. The EC‐RESC structure shows that GRBC components RESC1‐RESC4 are at some point ejected from this large complex that contains both mRNA and gRNA (Figure 2, Step 2). This coincides with gRNA transitions from the double stranded conformation it adopts in GRBC to a single‐stranded conformation in EC‐RESC. This transition permits gRNA‐mRNA association beyond the EC‐RESC surface, and presumably interaction with RECC (Liu et al. 2023) (Figure 2, Step 3), and REH2C (Kumar et al. 2016) (Section 4). mRNA is continuously fed through RESC as it contacts each of the EC‐RESC proteins (Figure 2, Step 3 blue arrow). Key to the assembly of EC‐RESC is the RESC10 protein. RESC10 was previously identified as a RESC organizer that was uniquely necessary for the stability of the RESC5/6 heterodimer, and high throughput sequencing indicated that RESC10 is essential for the downstream actions of several RESC proteins (Dubey et al. 2021). These findings were explained by cryo‐EM images showing that RESC10 and RESC14 approximately replace the positions of RESC1/2 next to RESC5/6. The gRNA U‐tail and adjacent nucleotides are held by RESC5/6/10, allowing the guiding and anchor portions of the gRNA to hybridize with mRNA beyond EC‐RESC and editing to commence (Liu et al. 2023). A third complex identified by cryo‐EM comprises RESC5, 6, 8, 10, and 14 and gRNA (Liu et al. 2023) (Figure 2, Step 4). A possible function of this complex is as a disassembly intermediate following complete utilization of a given gRNA. Disassembly of these proteins and a gRNA would then leave the next, more 5′, REMC on the mRNA free for RESC14 binding and subsequent association of another GRBC‐gRNA complex stabilized by RESC8 interaction (Figure 2).

### 3.5 RESC Associations With Additional Mitochondrial Factors

In addition to directly binding gRNA and mRNA and transiently interacting with RECC via RNA contacts, RESC associates with REH2C through mRNA:gRNA base‐pairing as proposed in Section 4 (Kumar et al. 2016), and with numerous auxiliary factors, several of which play transcript‐specific roles in editing. These include the RNA‐binding proteins KMRP1/2 (Weng et al. 2008), KRGG1 (Carnes et al. 2023; Hashimi et al. 2008), KRBP72 (Dubey et al. 2025), and DRBD18 (Pandey et al. 2025), as well as the RNA helicase KREH1 (Dubey et al. 2023) and the C1QBP homolog, p22 (Sprehe et al. 2010). The mRNA stability factor, KRGG3, appears to interact with RESC1/2 apart from the rest of RESC (McAdams et al. 2015). Interestingly, depletion of RESC12/12A hinders mRNA association of MRP1, KRGG1, and KRBP72 with mRNA, suggesting that one function of this REMC factor, or REMC in general, is to facilitate mRNA association of editing auxiliary factors (Dixit et al. 2017; Dubey et al. 2025). RESC has also been implicated in circularization of mRNA during the editing process through simultaneous interactions with the 3′ end associated polyadenylation machinery and the 5′ end associated PPsome (5′ pyrophosphate processome) (Mesitov et al. 2019). This RESC‐mediated mRNA circularization was proposed to ensure that only polyadenylated mRNAs undergo editing and to promote mRNA stabilization.

## 4 RNA Editing Helicase 2 Complexes

### 4.1 Discovery and Definition of REH2C

**FIGURE 3:**
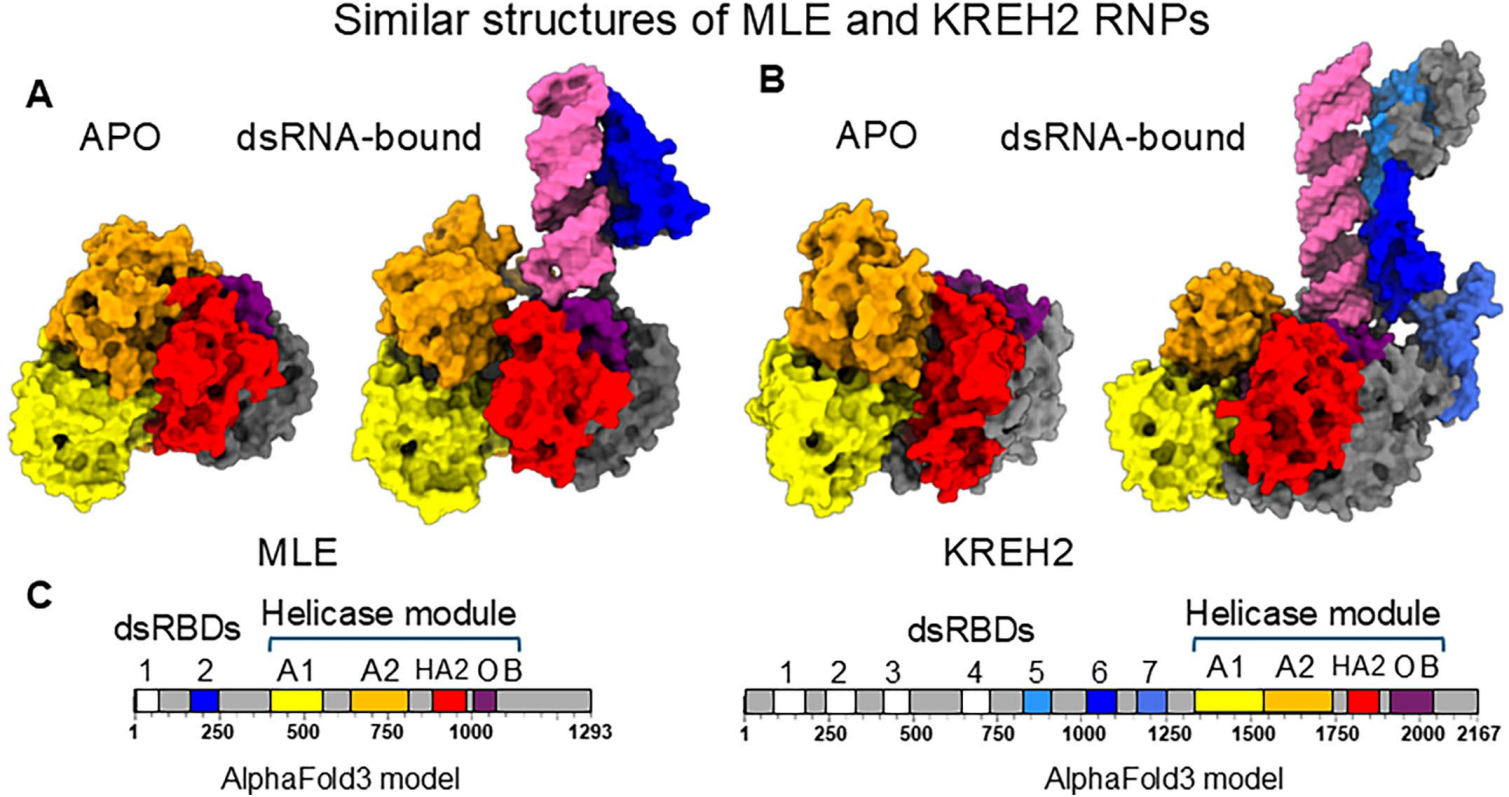
Structural similarity between MLE and KREH2 RNPs. (A) Cryo‐EM structures of APO (PDB: 8B9L) and dsRNA‐bound (PDB: 8B9K) MLE (Jagtap et al. 2023). (B) AlphaFold3 models of APO and dsRNA‐bound KREH2. (C) Domains of MLE and KREH2 are color‐coded and dsRNA is shown in pink.

REH2C (also known as RNA Editing Helicase KREH2‐Associated Complex) was identified in 2016 as an essential nucleoside triphosphate‐dependent molecular motor that unwinds dsRNA (Kumar et al. 2016). REH2C was proposed to modulate the assembly of the editing holoenzyme, substrate specificity, and editing fidelity, and recently emerged as a key factor in stage‐ and substrate‐specific regulation of editing during development in *T. brucei* (Cruz‐Reyes et al. 2016, 2018; Kumar et al. 2020, 2016; Meehan et al. 2024, 2023). REH2C represents mRNA‐associated ribonucleoprotein complexes (RNPs) isolated from mitochondria lacking gRNA‐bound RESC2 (Kumar et al. 2016). The REH2C RNPs contain editing substrates, intermediates, and products, and three main core proteins: KREH2 RNA helicase with ATP‐dependent 3′‐5′ unwinding activity, KH2F1 with eight C2H2 ZnFs, and KH2F2 with a likely inactive hydratase domain (Kinetoplastid KREH2‐Associated Factors 1 and 2) (Table 1) (Hernandez et al. 2010; Kumar et al. 2016; Madina et al. 2015). KH2F1 and KH2F2 are the most frequent interacting factors of KREH2 in reciprocal pull‐downs and mass spectrometry analyses of mitochondrial extracts (Hernandez et al. 2010; Kumar et al. 2016). The direct interaction between the three core proteins in REH2C was confirmed in akinetoplastic bloodstream stage trypanosomes devoid of mitochondrial RNA (Meehan et al. 2024). KREH2 shows a typical wide distribution in sedimentation gradients of mitochondrial extracts (Hernandez et al. 2010). In contrast, the KREH2 sedimentation is reduced to a narrow ~15 s peak upon depletion of mitochondrial RNA polymerase or RNase treatments, suggesting that the RNA‐free REH2C core is ~0.5 MDa in size (Hernandez et al. 2010; Kumar et al. 2016; Madina et al. 2015). So far, native or recombinant KREH2 has been reported to crosslink to RNA via UV irradiation (Hernandez et al. 2010; Kumar et al. 2016; Madina et al. 2015). KREH2 and the related *Drosophila* MLE and human DHX9 are DEAH‐box members of the helicase superfamily 2 (SF2), characterized by a conserved C‐terminal domain cluster that includes the helicase module and a regulatory OB‐fold (Jagtap et al. 2023). The N‐terminus of these three helicases contains tandem dsRBD motifs. Typically, RNA helicases have low specificity and require partner proteins that guide them to specific target substrates and locations (Studer et al. 2020). MLE and human DHX9 lack well‐defined cofactors, making REH2C particularly useful for studying mechanisms of cofactor‐mediated specificity. Interestingly, MLE structures revealed RNA‐induced autoregulation, involving alternate conformations: a dsRNA‐bound open conformation, in which the dsRBD2 domain aligns the substrate RNA with the helicase tunnel, and an ATP‐dependent apo‐closed conformation, in which dsRBD2 associates with the helicase module, blocking the tunnel (Jagtap et al. 2023). AlphaFold3 predictions of KREH2, both apo or dsRNA‐bound, suggest alternate conformations that resemble those in MLE (Figure 3), suggesting an autoregulatory dsRBD‐mediated function in helicase KREH2.

The composition and conformation of REH2C RNPs also appear to be dynamic. First, KREH2 associates with KH2F1 in the absence of KH2F2, and vice versa, both when using recombinant proteins and in vivo; however, KH2F1 and KH2F2 do not interact without KREH2. Second, core proteins in REH2C co‐purify but also show heterogeneous distribution in sedimentation gradients, indicating that these proteins are not always found together (Madina et al. 2015). Third, weak or transient protein partners seem to appear at relatively low frequency in reciprocal mass spectrometry‐pulldown studies of REH2C core proteins (Hernandez et al. 2010; Kumar et al. 2016) (unpublished data). Therefore, stable core REH2C RNPs may have transient and infrequent partner proteins. Furthermore, purifications of native REH2C revealed RNA‐mediated associations with RESC variants, RECC complexes, ribosomes, and other factors involved in gRNA or mRNA processing and maturation in mitochondria (Hernandez et al. 2010; Kumar et al. 2016; Madina et al. 2014, 2015; Meehan et al. 2024, 2023). In the procyclic stage, KH2F1 stabilizes KREH2 at steady state, serves as an adaptor that connects REH2C with RESC, and promotes RESC co‐purification with RECC (Kumar et al. 2016). Notably, an inactivating point mutation of the ATP‐binding motif I (MotI) and deletion or point mutation of dsRBD6 in KREH2 inhibit its association with RESC and RECC (Hernandez et al. 2010; Kumar et al. 2019; Madina et al. 2015). These findings indicate that the assembly of the editing holoenzyme, including productive contacts between REH2C, RESC, and RECC, requires ATP‐dependent remodeling by REH2C. Below, we first discuss (a) a docking model of REH2C and RESC via mRNA:gRNA hybridization, including proposed roles of REH2C in hybrid specificity and remodeling before, during, and after editing, and (b) a REH2C‐dependent model of developmental editing regulation.

### 4.2 Association of REH2C‐RESC via mRNA:gRNA Base Pairing, and Proposed Roles in Substrate Recognition and Editing

**FIGURE 4:**
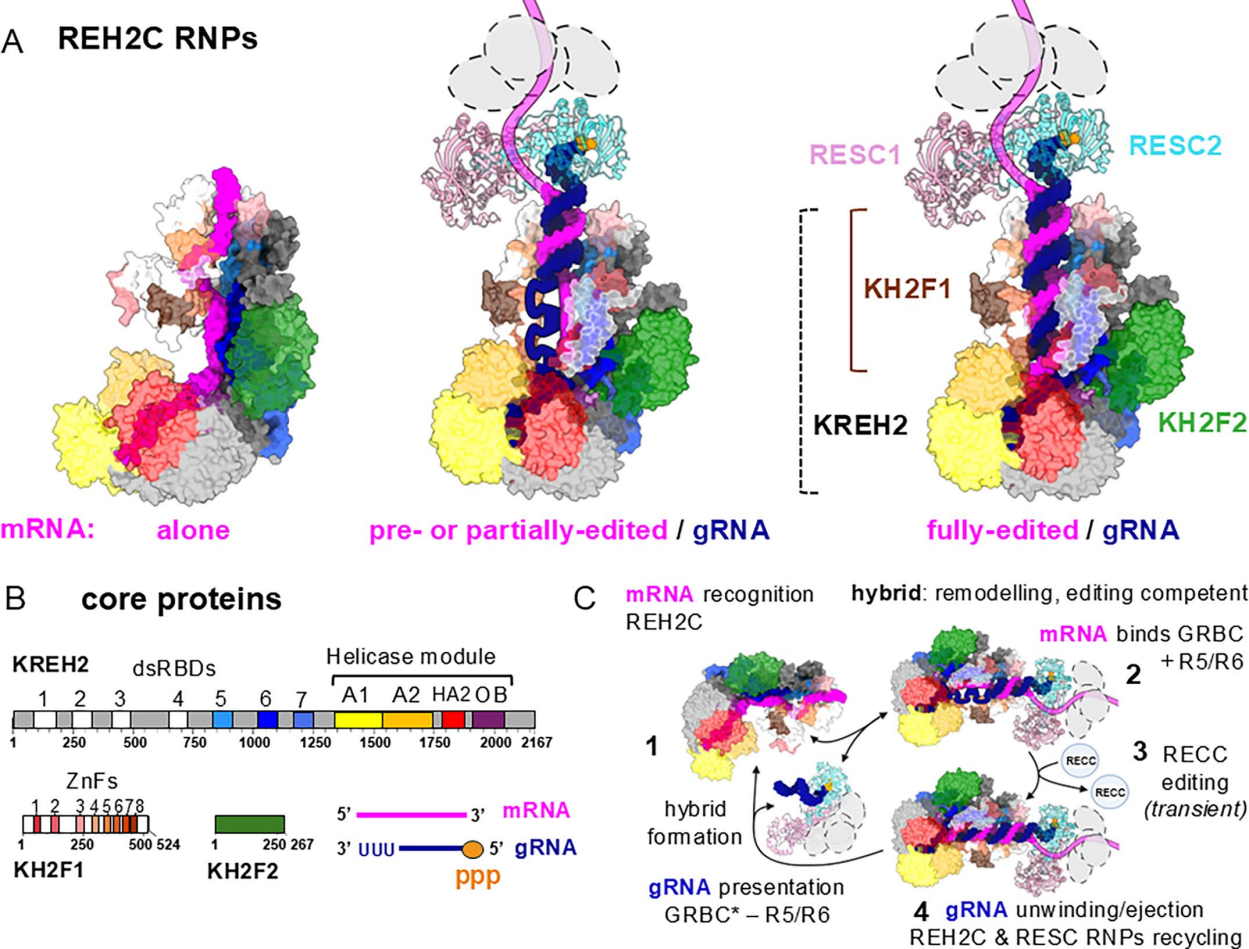
REH2C association with RESC via mRNA:gRNA base pairing. (A) Predicted structures of core REH2C (AlphaFold3) folded with mRNA alone or REH2C base‐paired with RESC1/RESC2 heterodimer (Boltz‐1) through an mRNA:gRNA hybrid in different stages of mRNA maturation: pre‐edited (left), partially edited (center), fully edited (right). RESC2 binding of gRNA 5′ triphosphate (gold) is also modeled. Gray circles symbolize other possible associated proteins in variants of RESC. See main text for details. (B) Domain organization of REH2C core proteins. mRNA (magenta) and gRNA (navy) with 5′ triphosphate (ppp; gold). (C) Model of REH2C hybridization with RESC variants and roles of the association (Kumar et al. 2016): Step 1: REH2C recognizes mRNA while the RESC* variant (lacking RESC5‐6 heterodimers) holds the gRNA 5′ end and exposes its anchor sequence for base pairing; Step 2: Upon hybridization of the RNPs, REH2C‐mediated “quality‐control” stabilizes or unwinds “discards” the mRNA:gRNA hybrid. RESC5‐6 heterodimers, other RESC proteins, and mRNA bind the editing‐competent RESC variant; Step 3: RECC‐catalyzed U‐indels extend the hybrid via numerous transient contacts. Step 4: REH2C unwinds the fully edited hybrid, ejecting gRNA‐RESC. A new gRNA‐RESC* can now hybridize as editing progresses. ATP‐mediated REH2C remodeling of RNAs or their RNPs may occur at all steps.

A docking model between REH2C and RESC variants proposed that these RNPs hybridize via their respective mRNA and gRNA cargos (Figure 4A,B) (Cruz‐Reyes et al. 2018; Kumar et al. 2019, 2020, 2016). REH2C RNPs retain mRNA following the loss of gRNA in RESC2 RNAi knockdowns (Kumar et al. 2016). REH2C RNPs accumulate pre‐edited and partially edited mRNAs, whereas fully edited mRNAs decrease as expected upon RESC2 loss (Kumar et al. 2016). Interestingly, native KREH2 and RESC6 immunoprecipitations using affinity‐purified antibodies revealed RESC variants: the RESC* (alias GRBC*), which lacks RESC6, and the standard RESC (alias GRBC), which contains RESC6. The purified core REH2C complex was stably associated with the RESC* variant, but the RESC6‐purified RESC variant exhibited a relatively weaker association with KREH2. These purifications were also tested for RESC13, a protein in the REMC module of RESC, indicating that this module can bind the RESC variants. RESC5 and RESC6 are usually found as heterodimers (see Section 3). Thus, RESC and RESC* may represent alternative configurations, with variable content of RESC5‐RESC6 heterodimers, and possibly other proteins in RESC. As expected, fully edited mRNA and RECC are enriched in RESC compared with RESC* (Cruz‐Reyes et al. 2018; Madina et al. 2014). In the docking model, the stable REH2C‐dsRNA‐RESC* interaction could play important roles before editing, in pre‐initiation complexes, whereas the relatively transient REH2C‐dsRNA‐RESC interaction may occur in the active editing holoenzyme, including RECCs (Figure 4C).

We updated the docking model and predicted structures for REH2C‐RNA‐RESC variant assemblies based on recent structures of MLE and DHX9 and current information on the editing process. As mentioned above, in the MLE structure and the KREH2 RNP model, dsRBDs are aligned with the RNA (Figure 3). A cryo‐EM reconstitution of the RESC1‐RESC2‐gRNA subcomplex showed that RESC2 holds gRNA via specific binding of its 5′ triphosphate group (see Section 3) (Dolce et al. 2023). Also, cryo‐EM reconstituted RESC variants, termed RESC‐B and RESC‐C, bind mRNA and gRNA; however, the two transcripts do not show base‐pairing within those structures. Instead, mRNA and gRNA regions outside RESC would be free to hybridize and associate with other factors, including REH2C and RECC (Liu et al. 2023). Predicted structures with AlphaFold3 and Boltz‐1, including the core REH2C with ssRNA, or the core REH2C with dsRNA, RESC1, and RESC2, may represent REH2C with mRNA alone or REH2C‐RESC association via mRNA:gRNA hybrids (Figure 4A). In the predicted structures, REH2C may play crucial roles before, during, and after editing. In the docking model, REH2C may specifically recognize editing mRNA transcripts through combinations of dsRBD and ZnF contacts (Figure 4C, Step 1). Structural features rather than base sequence may be recognized. In particular, the tandem array of eight C2H2 ZnF domains in KH2F1 may confer complex specificity in RNA recognition, as in other systems (Bohn et al. 2024; Cruz‐Reyes et al. 2016). gRNA‐RESC* lacking RESC5‐RESC6 dimers hold and present gRNA for potential hybridization (Figure 4C, Step 1). gRNA‐RESC* targets mRNA‐REH2C via gRNA anchor‐mediated annealing (Figure 4C, Step 2). REH2C may select and further stabilize high‐affinity “seed” duplexes after “quality‐control” contacts, probing the specificity of the RNA pair. Low‐quality duplexes would be unwound and “discarded”, dissociating REH2C‐RESC*, similarly to RNA‐targeting mechanisms by CRISPR‐Cas and the microprocessor in RNA silencing (Medley et al. 2021; Wang and Doudna 2023). Further interaction may include RESC5‐RESC6 and other RESC proteins. The new resulting RESC variant may bind mRNA in editing‐competent complexes (Figure 4C, Step 2). REH2C may remodel the hybrids to facilitate transient RECC contacts and countless rounds of editing on the pre‐assembled hybrids in the holoenzyme (Figure 4C, Step 3). Finally, REH2C may unwind the hybrid upon completion of the editing block, allowing a new gRNA‐RESC* to assemble (“gRNA exchange”) and proceed to edit the next block (Figure 4C, Step 4). Thus, REH2C may facilitate substrate recognition, ensuring that RESC* and RESC (including the RESC‐B variant) bind the correct mRNA. Since REH2C interacts with pre‐edited, partially‐edited, and fully edited mRNAs, and because REH2C association with RESC requires ATP, REH2C might remodel RNA hybrids at all stages of the process, including the exchange of gRNA‐RESC* during editing progression (Cruz‐Reyes et al. 2018; Kumar et al. 2016).

### 4.3 Developmental Regulation of Editing by REH2C

**FIGURE 5:**
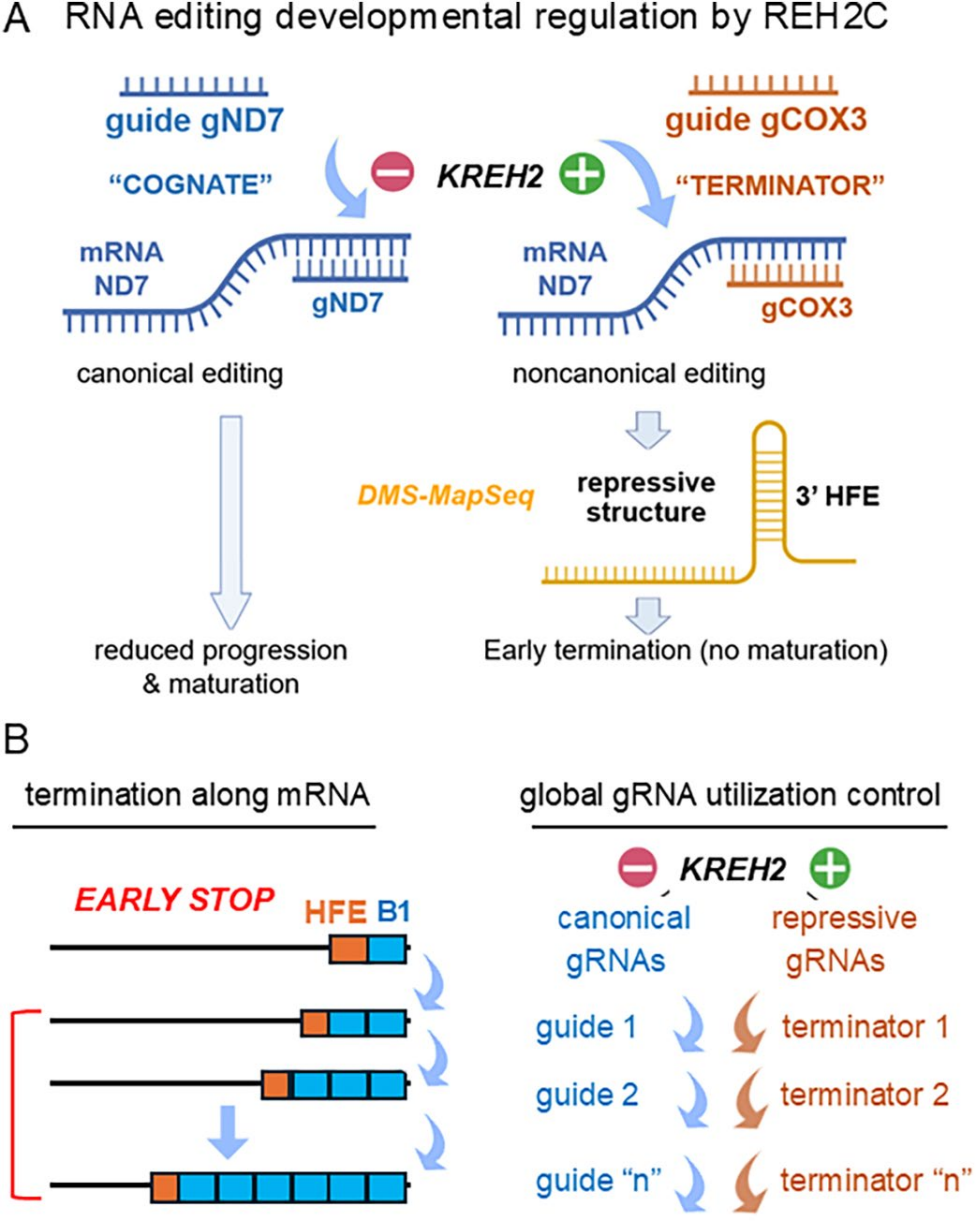
(A) Procyclic‐specific editing repression in ND7 mostly targets early editing and requires REH2C. Inhibition of editing involves concurrent negative and positive control of canonical (gND7) and terminator (moonlighting gCOX3) gRNA utilization. The terminator gRNA introduces a high‐frequency element (HFE) via non‐canonical editing, forming a repressive structure that may attenuate editing complexes (Meehan et al. 2024). (B) While most REH2C‐dependent repression targets early editing, this complex may also regulate gRNA utilization for canonical and programmed non‐canonical editing globally for maximal inhibition of specific substrates during development. See main text for details.

RNA editing is developmentally regulated between the procyclic and bloodstream stages of *T. brucei* ; however, it was only recently discovered that REH2C is a long‐sought key factor in stage‐ and substrate‐specific regulation of mitochondrial mRNA editing (Meehan et al. 2024, 2023). Editing proteins are required for mRNA maturation by definition; however, proteins in REH2C have a dual role: promoting general editing and mediating stage‐ and transcript‐specific editing repression during development. A model of REH2C‐dependent editing repression in the procyclic stage has two main features. First, most repression targets early editing and involves differential regulation of canonical and novel “moonlighting” gRNAs (Figure 5A). Second, moonlighting gRNAs introduce non‐canonical high‐frequency elements (HFEs) that block canonical editing and ultimately all editing action. Even 3′ terminal encoded bases not classified as part of the guiding domain in canonical editing can direct abundant non‐canonical edits in a REH2C‐regulated manner, as demonstrated for two initiator gRNAs. Such specialized non‐canonical edits by canonical gRNAs effectively “destroy” the binding site of the second canonical gRNA, promoting early termination (Kumar et al. 2020; Meehan et al. 2024). Non‐canonical editing differs from the canonical editing pattern required for mRNA maturation. Interestingly, HFEs may adopt an RNA conformation that attenuates editing complexes (Figure 5A). Procyclic‐specific repression by proteins in REH2C was first observed in two mRNAs, namely ND7, which encodes a subunit of respiratory complex I (NADH dehydrogenase), and A6, which encodes a subunit of the F_1_F_O_‐ATPase complex (complex V) (Meehan et al. 2024, 2023). However, the bloodstream stage‐specific repression of cytochrome‐encoding mRNAs also requires proteins in REH2C (unpublished data). Notably, induced in vitro differentiation from mammalian to insect stage of *T. brucei* , including pleomorphic strains, recreated the developmental regulation of canonical and alternative “moonlighting” gRNA utilization (Meehan et al. 2024, 2023). Pleomorphic strains are pre‐adapted to infection of the tsetse fly midgut (Matthews 2021). While stage‐specific REH2C‐dependent repression mainly impacts early editing, REH2C also appears to broadly regulate the use of the gRNA transcriptome to repress specific mRNAs during development—the putative “repressome.”

ND7 mRNA editing‐mediated maturation occurs mostly in the bloodstream stages (Koslowsky et al. 1990, 1992). Targeted amplicon‐PCR studies showed procyclic‐specific repression of ND7 editing that requires KREH2 and KH2F1. Most ND7 repression occurs early and involves simultaneous positive and negative regulation of moonlighting and canonical gRNA utilization, respectively (Figure 5A) (Meehan et al. 2024). HFE‐containing ND7 carries canonical edits downstream of HFE but not upstream and accounts for about 30% of the ND7 transcriptome in RESC6‐containing RESC complexes in the procyclic forms. This suggests that repression occurs within active editing holoenzyme. HFE‐bearing ND7 transcripts are rare in the bloodstream stages, where ND7 maturation is efficient. In fact, the moonlighting gRNA is bifunctional: it acts as a canonical gRNA in COX3 editing, and it also moonlights as a “terminator” in the non‐cognate ND7, where it adds a repressive HFE to halt early canonical editing. Analysis of HFE‐bearing ND7 mRNA structure in vitro using DMS‐MapSeq indicates that HFE causes an allosteric block that prevents downstream “repair” editing (Figure 5A), which explains the significant accumulation in procyclic forms. Remarkably, KREH2 depletion reversed the ND7 repression phenotype in procyclic forms, concurrently increasing and decreasing the use of canonical and terminator gRNAs, respectively (Meehan et al. 2024). A reversal of canonical and terminator‐directed non‐canonical editing was also observed throughout ND7, indicating that REH2C globally controls the use of the gRNA transcriptome during ND7 editing (Figure 5B).

A6 mRNA editing occurs in procyclic and bloodstream forms; however, A6 editing is also differentially regulated by proteins of REH2C (Kumar et al. 2020; Meehan et al. 2023). During A6 early editing, a moonlighting gRNA (i.e., a canonical guide in mRNA CR4 editing) introduces a repressive HFE. A6 mRNA transcripts with HFE lack any canonical edits, are enriched in RESC6‐containing RESC complexes, and are upregulated to over 30% of the A6 transcriptome after RNAi‐induced downregulation of KREH2 or KH2F1 in procyclic forms. Notably, downregulation of KREH2 or KH2F1 does not have this effect on the HFE in bloodstream forms (Meehan et al. 2023). Thus, A6 and ND7 exhibit notable differences in early regulation, including the moonlighting gRNA acting as an “anti‐initiator” in A6, preventing canonical initiation, or as a “terminator” in ND7, halting further canonical editing. DMS‐MapSeq analyses of HFE‐bearing A6 in vitro suggested that the HFE introduces an allosteric block in editing. The binding of moonlighting gRNAs to both cognate and non‐cognate mRNAs was confirmed in vivo by isolating chimeric mRNA:gRNA molecules (Meehan et al. 2024, 2023), which are editing byproducts but demonstrate on‐target association (Seiwert et al. 1996). Overall, studies of ND7 and A6 support a general model of substrate‐ and stage‐specific regulation by proteins in REH2C. In ND7, REH2C maintains procyclic‐specific repression; however, in A6, REH2C promotes efficient maturation in procyclic forms by preventing HFE‐mediated repression. While REH2C exerts most control in early editing, this complex may control the entire gRNA transcriptome in the studied substrates during development (Meehan et al. 2024, 2023). Target selection by canonical versus terminator gRNA enabling mRNA expression or repression, respectively, may occur during the second step in the REH2C‐dsRNA‐RESC* docking model, when RNA hybrids are either stabilized or discarded (Figure 4C).

### 4.4 Implications of Terminator‐Mediated Repression in Mitochondrial Energetics and the CNE Hypothesis

REH2C‐regulated terminators impact mitochondrial energetics, primarily during early editing, by installing abundant non‐canonical HFEs. For example, HFEs can occur in about 30% of the ND7 and A6 transcriptomes (Meehan et al. 2024, 2023). Based on the ATP hydrolysis required for RNA ligation at each editing site (see Section 2) (Cruz‐Reyes and Sollner‐Webb 1996; Kable et al. 1996; Seiwert et al. 1996; Seiwert and Stuart 1994), full editing of ND7 and A6 requires 14 or 30 times more ATP, respectively, than the formation of HFE to stop editing early. REH2C‐mediated termination and the potential allosteric block by structural HFEs are “energy‐saving” as they attenuate editing catalysis in specific mRNAs. This repression model, initially described in the procyclic forms, also operates and is more robust in the bloodstream stage‐specific editing repression (unpublished data). REH2C‐mediated control of cognate and non‐cognate gRNA utilization and, implicitly, energy consumption is a novel feature in *T. brucei* metabolism (Meehan et al. 2024, 2023). Finally, a popular evolutionary model posits that RNA editing is error‐prone and emerged through constructive neutral evolution (CNE) (Gray et al. 2010; Lukeš et al. 2011; Stoltzfus 1999) (Section 7). In the CNE model, some features, including non‐canonical editing, lack clear benefits and are neutrally fixed by genetic drift. However, the discovery that at least some non‐canonical HFEs are encoded and controlled by specific factors may be an exception. An alternative view is that the initial evolution of HFEs could have been neutral, but the later acquisition of specialized regulatable HFE functions was fixed by Darwinian‐positive selection (Meehan et al. 2024, 2023). Whatever the specific model, these observations nicely illustrate the intricate interactions between neutral and possible selective aspects of RNA editing (Section 7).

## 5 Computational Mapping of RNA Editing Complex Protein Interactions in Trypanosomatid Pathogens

As discussed above, the RNA editing process depends on dynamic PPIs both within and between RECCs, RESC, REH2C, and other editing auxiliary factors (Table 1), and these dynamics may change during the developmental regulation of editing between diverse host and vector environments (Carnes et al. 2022; Davidge et al. 2023; McDermott, Carnes, and Stuart 2015; McDermott et al. 2019, 2024; McDermott, Guo, et al. 2015; McDermott and Stuart 2017; Meehan et al. 2024, 2023; Zamani et al. 2024). Understanding the detailed dynamic molecular interactions that occur during RNA editing is particularly critical, as this process and the machinery that carries it out are unique to kinetoplastids, making them a potential therapeutic vulnerability for the diseases caused by the pathogenic parasites in this group. Due to experimental limitations and limited homology with other model organisms, computational approaches are critical for mapping their PPI networks. Advances in artificial intelligence, high‐resolution structural modeling, and systems‐level network biology have shifted the field beyond homology‐based inference toward integrative, data‐driven prediction frameworks. These methods not only improve our understanding of editing complex dynamics but also generate experimentally testable hypotheses with direct relevance to therapeutic discovery. By comparing classical approaches with cutting‐edge strategies, we emphasize the power of hybrid pipelines that integrate structural, functional, and regulatory information to chart new directions in kinetoplastid mitochondrial RNA biology.

### 5.1 Rethinking Classical Approaches

#### 5.1.1 Sequence‐Based Inference: Strengths and Limitations

Traditional protein function prediction often relies on sequence homology, yet several “nonhomology” computational methods, such as domain fusion, conserved gene order, phylogenetic profiles, and co‐expression, that predict protein function and potential interactions from genomic context rather than overall sequence similarity have also been described (Altschul et al. 1997; Marcotte 2000; Smith and Waterman 1981). However, these approaches still require initial sequence homology searches (e.g., BLAST) to identify orthologous proteins or domains across species. While effective for model organisms, these methods prove inferior for kinetoplastid proteins due to their divergent sequences and domain structures, leaving many proteins without annotated orthologs (Salavati and Najafabadi 2010). Nevertheless, sequence‐based tools still have value, for example, PANNZER2 was used to re‐annotate the *T. brucei* proteome, assigning GO terms to uncharacterized proteins (Borujeni and Salavati 2024b). Other studies have highlighted the value of sensitive profile‐profile alignment tools, such as HHpred, for inferring the functions of highly diverged domains, including those in editing complex components (Carnes et al. 2018; McDermott, Carnes, and Stuart 2015; McDermott et al. 2019, 2016; McDermott, Guo, et al. 2015; McDermott and Stuart 2017; Meehan et al. 2024; Nikpour and Salavati 2019). Integrating such profile‐based methods with domain co‐occurrence data (patterns where specific domains are repeatedly found together within the same protein or across interacting proteins) can substantially improve the identification of protein interaction partners within editing complexes. In addition, genes encoding interacting proteins often co‐evolve, producing similar phylogenetic histories. Thus, resemblance between the phylogenetic trees of two genes can indicate physical or functional interaction (Pazos and Valencia 2001). In the specific context of editing complexes, sequence‐ and profile‐based strategies are most powerful when anchored to validated RECC, RESC, and REH2C components. These known subunits serve as effective “baits” for detecting kinetoplastid‐specific interaction partners via domain co‐occurrence, phylogenetic profiling, and co‐evolutionary analysis. Deep profile‐based tools (e.g., HHpred, HHblits) remain useful for identifying cryptic domains and recurrent motif combinations reflecting subcomplex modularity. Relatedly, phylogenetic trees reconstructed from pre‐edited mitochondrial transcripts can be compared with those of editing proteins to assess RNA–protein co‐adaptation. Still, the abundance of low‐complexity regions, paralogs, and poor synteny reduces the resolution of purely sequence‐based methods. These approaches are most informative when applied to prioritized subsets, such as mitochondrially enriched or stage‐regulated proteins and integrated with orthogonal data from localization, genetic interactions, or structural models (e.g., AlphaFold‐Multimer).

#### 5.1.2 Structural Modeling and Comparisons

Structure‐based prediction via homology modeling and docking tools (e.g., HADDOCK, ClusPro) remains key for assessing biophysical interaction potential (Dominguez et al. 2003; Kozakov et al. 2017). However, accuracy is often limited when applied in kinetoplastids, for which there are sparse structural data and a lack of close templates, that both render homology models unreliable. Integrating complementary data, such as cross‐linking, which is available for RECC proteins (McDermott et al. 2016), or cryo‐EM structures (Liu et al. 2023) will substantially improve prediction quality. While docking approaches focus on modeling interactions between specific protein pairs, broader comparative strategies that align structures across proteins or species can provide insights into conserved and variable interaction interfaces, offering a deeper understanding of their evolutionary and functional variation. Accordingly, structural comparison for broader interface mapping can complement sequence‐based annotation by revealing features directly relevant to interaction specificity. Pairwise structural similarity tools such as Foldseek (Kim et al. 2025), Reseek (Edgar 2024), and motif‐centric search engines like Folddisco (Kim et al. 2025) can detect complementary interface geometries or 3D motifs that sequence‐only methods often miss, particularly in highly diverged editing complex proteins (Borujeni and Salavati 2024a). Extending this to multiple orthologs or paralogs through multiple structure‐based alignments with tools like US‐align (Zhang et al. 2022), FoldMason (Gilchrist et al. 2024), or MUSCLE‐3D (Edgar and Tolstoy 2024) enables the identification of conserved cores and variable regions across taxa. Such conservation mapping can highlight residues maintained across species that are critical for interface formation, while pinpointing lineage‐specific alterations that may underlie functional divergence in PPIs. For editing complexes, structure‐centered approaches are particularly valuable for rationalizing how diverse subunits assemble into modular machines and how distinct complexes interface with one another and with RNA substrates. Comparative analysis of available and emerging structures for RECC, RESC, and associated helicase or auxiliary modules can reveal recurring interface geometries and conserved surface patches that are not apparent from sequence alone, and that may correspond to docking sites for gRNA, pre‐mRNA, or partner complexes. Multiple structure alignments across kinetoplastid species, or between paralogous subunits within a species, could further pinpoint lineage‐specific insertions or conformational shifts that underlie differences in editing efficiency, substrate preference, or regulation. In practice, these structural comparisons will be most powerful when combined with mutagenesis, cross‐linking, and functional assays to distinguish interfaces that are essential for complex integrity from those that tune editing dynamics or complex–complex crosstalk.

### 5.2 Emerging Frontiers

#### 5.2.1 AI Models Redefining PPI and Protein‐Ligand Prediction

The arrival of AlphaFold2 and its multimer extension have reshaped computational structural biology (Evans et al. 2021), and have provided structural predictions for several editing complex subunits for which direct structural information is lacking (Carnes et al. 2022; Davidge et al. 2023; McDermott et al. 2024). However, AlphaFold requires a high‐quality Multiple Sequence Alignment (MSA) for accurate structure prediction (Jumper et al. 2021), which might be challenging to create for editing complex subunits as they have few homologs outside the kinetoplastid lineage. Incorporating organism‐specific sequence databases into the default AlphaFold pipeline can partially mitigate this limitation and substantially improve predictions for orphan proteins (Wheeler 2021). Moreover, emerging alternatives such as ESMFold (Hayes et al. 2025) and Chai‐1 (Discovery et al. 2024) leverage protein language models to predict structures directly from individual sequences. By eliminating the MSA requirement, these methods are well‐suited for proteins with few or no homologs—common among editing complex subunits—while still achieving accuracy comparable to traditional MSA‐based pipelines. In addition to protein–protein assemblies, emerging AI models such as AlphaFold3 (Abramson et al. 2024) and Boltz (Passaro et al. 2025) can additionally co‐model protein–RNA–protein complexes, which is especially relevant for RNA‐mediated PPIs in kinetoplastids, and as discussed previously, were used to model REH2C‐dsRNA‐RESC assemblies (see Section 4; Figure 4). Explicitly incorporating RNA into these predictions may refine interface geometry, improve subunit placement, and reveal RNA‐dependent interaction surfaces that are otherwise challenging to resolve. This capability extends the application of AI models from binary protein interactions to multi‐component assemblies, expanding the scope of computational PPI mapping in RNA editing complexes. Beyond complex assembly, these models can also co‐fold proteins with alternative small molecules, enabling in silico screening for binding compatibility and specificity. Such screens can generate hypotheses about ligand‐ or RNA‐dependent functions, suggest regulatory mechanisms, and prioritize small molecules or oligonucleotides for experimental validation, or also as potential therapeutic candidates.

AI approaches are also beginning to address conformational dynamics, providing insight into transient or alternative states that influence PPIs. Modifying MSA inputs to AlphaFold or using ensemble‐prediction models such as BioEMU‐1 (Savaş et al. 2025) or ESMFlow (Frank et al. 2024) can yield plausible alternative conformations. These states may expose, occlude, or remodel interaction interfaces, offering hypotheses for stage‐specific or condition‐dependent PPIs in editosomes. Incorporating dynamics into AI‐based predictions adds a temporal dimension to PPI mapping, moving beyond static structures toward models that capture the full spectrum of interaction possibilities.

#### 5.2.2 Multi‐Omic Integration and Network‐Centric Approaches

Recent advances in systems biology and AI modeling have furthered understanding of RNA editing in *T. brucei* by integrating transcriptomic, proteomic, and functional data. As explored in previous sections, trypanosome mitochondrial RNA editing is governed by intricate networks of protein complexes, including RECCs, RESCs, and REH2Cs. Integrating such PPI networks with other layers of biological data, such as gene co‐expression and regulatory networks, holds significant potential for uncovering functionally relevant interactions and underlying regulatory mechanisms (Salavati and Gazestani 2019). Such integrative network approaches can provide context to physical interactions by revealing whether interacting proteins are co‐regulated or co‐expressed under specific conditions or developmental stages. For example, the expression levels of protein subunits within and associated with editing complexes were shown to be differentially regulated across life cycle stages using proteomic profiling of *T. brucei* mammalian bloodstream form and insect procyclic stages differentiation (Zamani et al. 2024). Some proteins that interact with the editing machinery, such as RBP7910 and KREH1, were upregulated during differentiation and in the insect stage, potentially indicating roles in developmentally regulated editing. This study underscores how multi‐omics approaches can reveal stage‐specific remodeling of RNA editing machinery when layered onto PPI maps. Modeling such transitions and expanding models to incorporate additional network layers may also help identify regulatory nodes—for example, kinases or post‐translational modifications—that modulate activity beyond expression levels.

**FIGURE 6:**
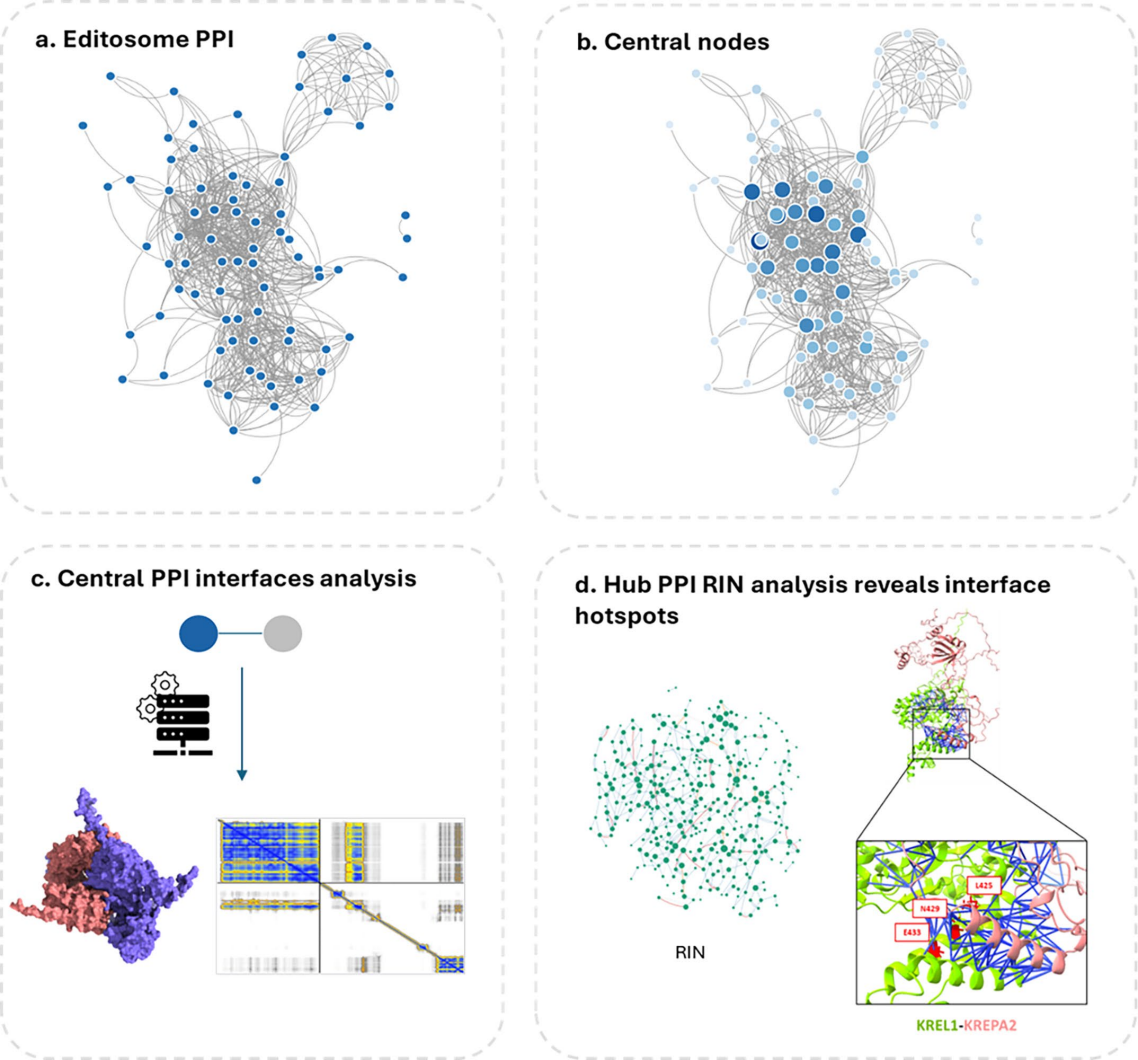
Network‐based and residue‐level analysis pipeline of protein–protein interaction interfaces of editing complexes in *T. brucei* , based on Poorinmohammad and Salavati (2024).

On a more granular level, Protein Interaction Networks (PINs) capture emergent properties such as redundancy, modularity, and centrality, which can highlight key PPIs, reveal critical regulatory hubs, and provide insight into the dynamic organization and functional architecture of protein complexes. In this context, a study by Poorinmohammad and Salavati (Poorinmohammad and Salavati 2024) integrated structural topology and network centrality metrics to systematically rank central editosome proteins and map their interaction interfaces. By analyzing the editosome PIN alongside Residue Interaction Networks (RINs) for hub proteins, the study identified key residues and interfaces that may influence complex stability and function, thereby providing focused candidates for future functional validation and therapeutic exploration (Figure 6). For instance, two RECC ligases, L1 and L2, were among central PPIs whose interaction structure was modeled. The interface was analyzed using RIN, and hot spot residues were shown to be localized mainly in the C‐terminal regions with a single alpha helix domain in their interacting partners, KREPA1 and KREPA2 (Poorinmohammad and Salavati 2024). Such hybrid methods are especially valuable in systems with limited homology to other model organisms, helping identify functionally important, potentially druggable interfaces.

### 5.3 Computational Perspective

**FIGURE 7:**
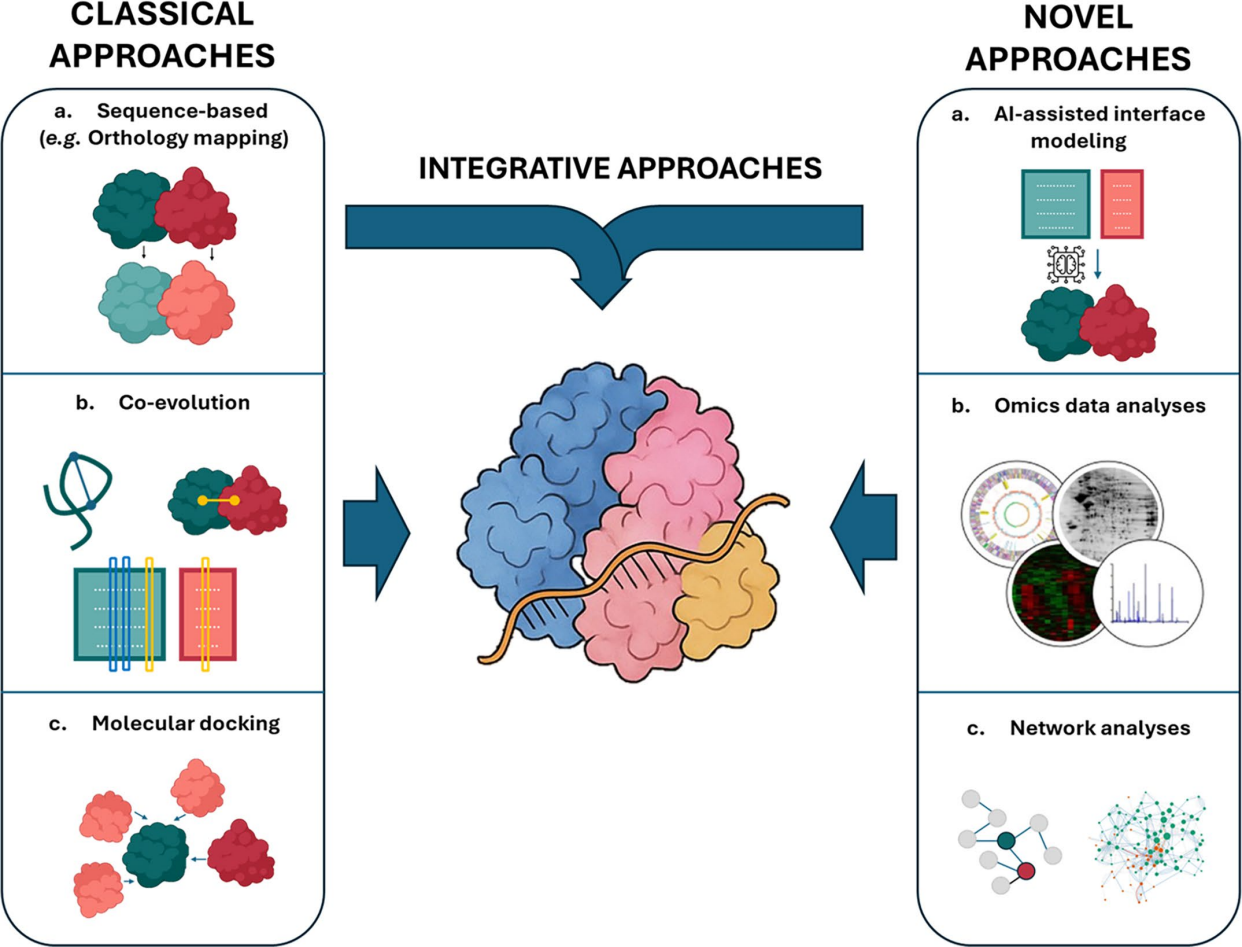
Schematic representation of predicting and modeling strategies for *Trypanosoma* editing protein–protein interactions.

Computational prediction of PPIs in kinetoplastids has evolved from isolated, algorithm‐driven approaches toward integrative frameworks that can incorporate AI‐based structure prediction, network analysis, and omics‐level data. Figure 7 summarizes the two approaches. While such models require experimental validation, approaches like that of Poorinmohammad and Salavati (2024), which integrate AlphaFold‐predicted structures with residue‐level network metrics, offer a promising and potentially generalizable framework for interactome analysis in kinetoplastids, as already shown for mitochondrial RNA editing complexes (see Section 4; Figures 3 and 4). Looking ahead, key priorities include generating curated, stage‐specific interaction maps to contextualize predictions and improving the interpretability of deep learning models—especially interface confidence and residue‐level insights. Expanding predictive pipelines to kinetoplastids beyond *T. brucei* could uncover conserved and divergent features. Incorporating dynamic elements such as post‐translational modifications and spatial–temporal regulation will enhance network models. Ultimately, an iterative cycle of prediction and experimental validation will drive a more complete systems‐level map of kinetoplastid mitochondrial RNA biology, with implications for understanding and targeting the neglected diseases these parasites cause.

## 6 RNA Editing Complexes and Guide RNA Repertoires Across Kinetoplastea

### 6.1 RNA Editing Complexes

A birds‐eye view of the U‐indel RNA editing machinery including RECCs, RESC, and REH2C reveals remarkable conservation across the highly divergent Kinetoplastea (see Section 7). Despite minor lineage‐specific instances of gene deletion or duplication among the trypanosomatid lineages (Afonin et al. 2024), a nearly 1:1 maintenance of editing factors exists. Comparatively, conservation of complexes that physically associate with editing machinery but that act in related activities may be more relaxed. For example, the *T. brucei* six‐subunit mitochondrial 3′ processome (MPsome) functions in RNA trimming (Suematsu et al. 2016). Only its catalytic subunits appear in *Perkinsela* (see Section 7), and while candidates for the noncatalytic subunits exist in more closely related species, these proteins share far less similarity than true editing factors between the same species (Afonin et al. 2024). Therefore, editing complexes stand out in uniformity, even compared with proteins of related processes.

Of course, even if present in all species, divergence of individual editing factors' features or functions could lead to species specificity in U‐indel RNA editing execution. The editing factors of *Blastocrithidia nonstop* provide insight into this possibility (Afonin et al. 2024). *B. nonstop* editing factors are ~30% longer than the *T. brucei* homologs due to multiple short insertions, likely due to the lack of a functional DNA non‐homologous end joining pathway (Nenarokova et al. 2019). However, when the *B. nonstop* RESC subunits were modeled on the *T. brucei* RESC structure (Liu et al. 2023), insertions were in positions unlikely to influence individual protein or complex structure (unpublished data). Similarly, the *B. nonstop* catalytic L1 ligase orthologue modeled on the crystal structure revealed little that would be predicted to interfere with its ligase activity. The apparent lack of insertions able to influence RESC or L1 functions suggests they are maintained by strong selective forces. Thus, profound functional alterations in individual editing factor subunits are unlikely.

### 6.2 Organization of Guide RNA Genes

**FIGURE 8:**
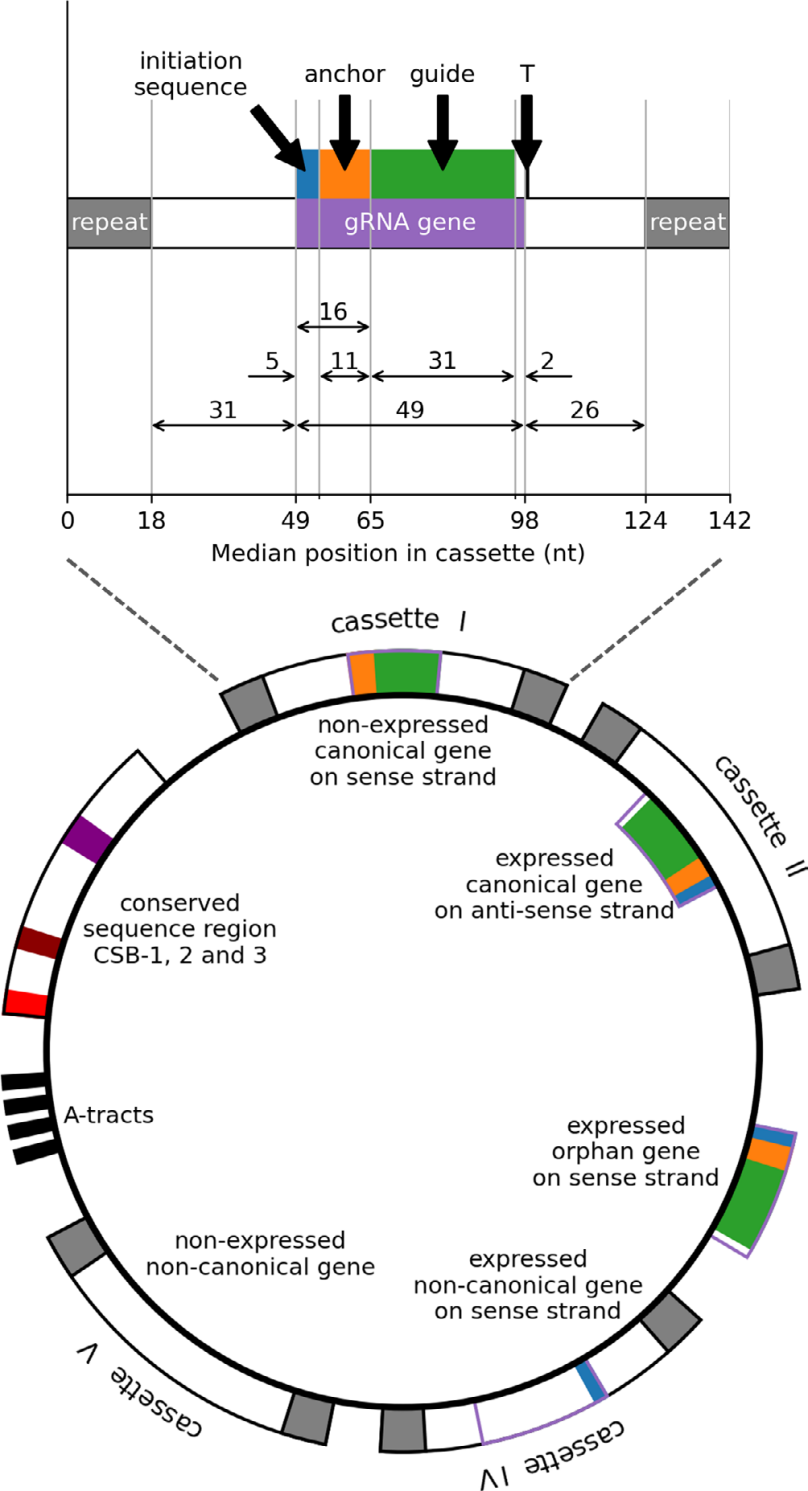
Schematic of a hypothetical *T. brucei* minicircle, with the structure of a typical gRNA gene cassette shown above. Four to five A‐tracts, each ∼5 bp long and positioned roughly in phase with the helical repeat, induce a bend in the minicircle. Downstream of this feature is the conserved sequence region with the highly conserved motifs CSB‐1 and CSB‐3 and the less well conserved motif CSB‐2. The major gRNA gene cassettes are labeled (in 5′ to 3′ order) I, II, IV, and V. A cassette III is sometimes found between cassettes II and IV. Cassettes are defined by a pair of imperfect 18‐bp repeats (dark gray segments). Canonical gRNA genes can be identified by alignment with edited mRNA and are usually encoded on the sense strand (here exemplified by cassettes I, IV, and orphan) or rarely on the antisense strand (exemplified by cassette II). Non‐canonical genes of uncertain functionality are identified by codon bias and conserved distance to the forward repeat (exemplified by cassettes IV and V). The schematic on top indicates median values for initiation sequence (blue), anchor region (orange), guiding region (green), and other distances with cassettes. Orphan gRNA genes are sometimes found encoded between cassettes II and IV on the sense strand. gRNA genes commonly end with a T nucleotide. Small transcriptome analysis allows determination of expression status. Data are from reference Cooper et al. (2022).

In contrast to the editing protein machinery, species variability abounds in genomic gRNA loci arrangements and gRNA repertoires. Across organisms, we encounter varied structures and complexities of gRNA‐encoding molecules, newly observable with sophisticated assemblies of high throughput sequence libraries (Afonin et al. 2024; Callejas‐Hernández et al. 2021; Cooper et al. 2019; Gerasimov et al. 2025, 2021; Gerasimov, Afonin, et al. 2022; Li et al. 2020; Simpson et al. 2015; Wang et al. 2025). Circular molecules of ~0.7–2 kb containing one or a few gRNA loci are common to mammalian‐pathogenic trypanosomatids and known as “minicircles”. However, minicircle features considered standard, such as size, are not universal across kinetoplastids (Table 2). For example, *Vickermania* spp. possess “HL‐circles” of 22 or 15 kb rather than minicircles (Gerasimov et al. 2025), and *Trypanoplasma borreli* kDNA contains seventeen ~70 kb linear contigs, each encoding 20–30 gRNAs per molecule (Gerasimov, Afonin, et al. 2022). Despite these differences, gRNA encoding molecules do share some common features. One is a region of strong sequence conservation among all the molecules within a species (the CR)—often also similar in sequence between species—and the variable sequence region (the VR) where gRNAs reside. Within minicircles, CRs include multiple A‐tracts, which induce bends in minicircle DNA, in addition to conserved sequence blocks (CSBs), whose conserved order and spacing indicate their ancestral and vital roles in minicircle replication (Figure 8) (Jensen and Englund 2012; Li et al. 2020).

**TABLE 2:** The RNA editing mechanism across Kinetoplastea.

Species	gRNA‐containing molecule characteristics				gRNA characteristics (Average)		
	Description	Number of classes^a^	gRNAs per molecule	gRNA genes^b^	Length (nt)	Mis‐match	G/U Pairs
*Trypanosoma brucei*	Circles of ~1000 bp, arranged in cassettes: [a gRNA + conserved domain containing CSB1‐3 and bend region]. gRNAs positioned between 18‐mer IRs.	391	3–4	1218	42	0.03	0.04
*Trypanosoma lewisi*	Circles of ~1290 or 1500 bp, with one or two conserved regions, one bend region, and a variable region containing gRNAs.	58	1–4	190	42–49^c^	N/A	N/A
*Leishmania tarentolae*	Circles of ~850 bp with a single conserved region and variable region. gRNAs positioned at a fixed distance from conserved domain element with a motif nearby.	114	1	114	43	0.06	0.2
*Leptomonas pyrrhocoris*	Circles of average size of ~1200 bp containing two conserved and two variable, gRNA containing regions. gRNAs located near 66‐mer motif.	67	1–2	107	32	0.1	0.2
*Blastocrithidia nonstop*	Circles of average size 2000 bp, similar to the structure of “dimeric” *L. pyrrhocoris* minicircles.	41	1	42	47^c^	N/A	N/A
*Vickermania spadyakhi*	“HL”‐circles of ~15,000 bp. Conserved regions present in multiple copies in four cassettes positioned in polar locations on circles. gRNAs flanked by inverted repeats	501	5	5033	N/A	N/A	N/A
*Vickermania ingenoplastis*	Similar to *V. spadyakhi* HL‐circles except longer (~22,500 bp) and four cassettes positioned in polar locations are present.	64	2	128	N/A	N/A	N/A
*Trypanoplasma borreli*	Likely linear molecules ~70 kb in length with repetitive termini.	17	23	420	28	0.13	0.11

*Note:* gRNAs encoded on the maxicircle are not included in totals. IR, Inverted Repeat. Data collected from {PMID: 36420151, PMID: 26204118, PMID: 40203041, PMID: 38452217, PMID: 33660779, PMID: 35414587, PMID: 31665448, PMID: 32853372}.

^a^ No consistent definition exists of the degree of homology that supports minicircles' belonging to a single class (Cooper. 2022 PMID: 35414587).

^b^ Putative gRNAs, not necessarily confirmed with small RNA sequencing.

^c^ Have been generated with different algorithms.

Guide RNA genes have often been identified by searching for sequence complementarity to canonically edited mRNA sequences. The structure and arrangement of such identified gRNA genes on their encoding molecules varies among kinetoplastids (Gerasimov et al. 2025; Li et al. 2020; Wang et al. 2025). For example, each minicircle encodes between three and five gRNAs in *Trypanosoma congolense*, *T. brucei* , and *Trypanosoma musculi* (Cooper et al. 2019; Pollard et al. 1990; Read and Stuart 1993; Wang et al. 2025). In *T. brucei* and *T. congolense*, gRNA genes typically reside on the sense strand in cassettes flanked by imperfectly conserved inverted repeats of 18 or 19 bp, respectively (Figure 8, cassette I). These repeats have been proposed to be involved in gRNA expression or maturation, but they might also serve as recombination hotspots between minicircles (Cooper et al. 2019, 2022; Jasmer and Stuart 1986; Pollard et al. 1990; Read and Stuart 1993; Suematsu et al. 2016). A few gRNA genes have also been predicted for the antisense strand of a cassette (Figure 8, cassette II) (Cooper et al. 2019). Both strands of minicircles are transcribed into approximately 800‐nt gRNA precursors which are processed into mature gRNAs by uridylation‐induced, antisense transcription‐controlled 3′‐5′ exonucleolytic degradation, providing a potential mechanism to produce both sense‐ and antisense‐strand encoded gRNAs (Suematsu et al. 2016). Not all minicircle cassettes contain sequences that can be aligned to the known edited mRNAs. Some cassettes contain predicted gRNA genes that are identified based on the nucleotide bias that is characteristic of gRNA genes, but that do not show sufficient complementarity to known edited mRNAs (Figure 8, cassettes IV and V). These 'cryptic' genes are often expressed, but it is unclear if the transcripts serve a function (Cooper et al. 2019, 2022). Interestingly, a few gRNA genes, including all gRNA genes needed for editing of the complex III subunit CYb mRNA, are found outside of cassettes, in the middle region of a minicircle, hence termed ‘orphan’ gRNA genes (Cooper et al. 2019; Hong and Simpson 2003; Riley et al. 1994). In contrast, minicircles in *Leishmania* spp. and *Crithidia fasciculata* encode only one gRNA and lack cassettes (Sturm and Simpson 1991; Yasuhira and Simpson 1995). In these cases, gRNA genes are typically found at conserved distances from specific minicircle features (Ramakrishnan et al. 2024; Simpson et al. 2015; Sturm and Simpson 1991; Van den Broeck et al. 2020; Yasuhira and Simpson 1995).

The number of different minicircle (or their equivalent) sequences that comprise a full assembly is also species‐specific, as is the copy number of the different sequences (Gerasimov et al. 2025, 2021; Koslowsky et al. 2014; Li et al. 2020; Simpson et al. 2015). Deep sequencing studies have identified up to 400 distinct minicircle sequence classes in *T. brucei* (Cooper et al. 2019, 2022; Geerts et al. 2022; Zhao et al. 2025), up to 200 in *Leishmania braziliensis* (Van den Broeck et al. 2020), 100–120 in *Leishmania major* and *Leishmania tarentolae* (Camacho et al. 2019; Simpson et al. 2015), 41 in *B. nonstop* (Afonin et al. 2024), 39 in *T. musculi* (Wang et al. 2025), and only 18 in a laboratory strain of *C. fasciculata* (Ramakrishnan et al. 2024). At the very extreme, dyskinetoplastic forms of *T. brucei* such as *T. b. evansi* and *T. b. equiperdum*, may contain only a single type of minicircle (Borst et al. 1987; Schnaufer et al. 2002). Non‐identical gRNA gene sequences encoded by distinct minicircle classes can often be grouped into clusters of functionally homologous gRNA sequences that align to closely overlapping regions of edited mRNA sequences, revealing substantial redundancy in their gRNA repertoires (Cooper et al. 2019, 2022; Kirby et al. 2016; Koslowsky et al. 2014; Rusman et al. 2021; Simpson et al. 2015; Zhao et al. 2025). Interestingly, numbers of minicircle sequence classes may also even vary within a species. For instance, recently isolated *Vickermania spadyakhi* possesses 501 sequences while long‐cultured *Vickermania ingenoplastis* has a mere 64. The former but not the latter is able to productively edit the respiratory complex I subunit‐encoding cryptogenes (Gerasimov et al. 2025). A remarkably similiar scenario had been reported much earlier for *L. tarentolae*, where extended cultivation in the laboratory also resulted in the loss of minicircles encoding gRNAs for complex I (Simpson et al. 2015; Thiemann et al. 1994). Thus, correlations exist between loss of specific gRNA‐containing molecules and the extent of editing of the maxicircle‐encoded mRNAs, depending on the metabolic needs of the individual species and/or due to long‐term growth in culture (Gerasimov, Afonin, et al. 2022; Simpson et al. 2015). No instance of partitioning of gRNA loci onto specific DNA molecules based on their cognate mRNAs has been found. Given this, the species diversity of gRNA‐containing molecule size, numbers, and gRNA loci per molecule provides an opportunity to elucidate the evolutionary benefits and disadvantages of all these relative metrics.

**FIGURE 9:**
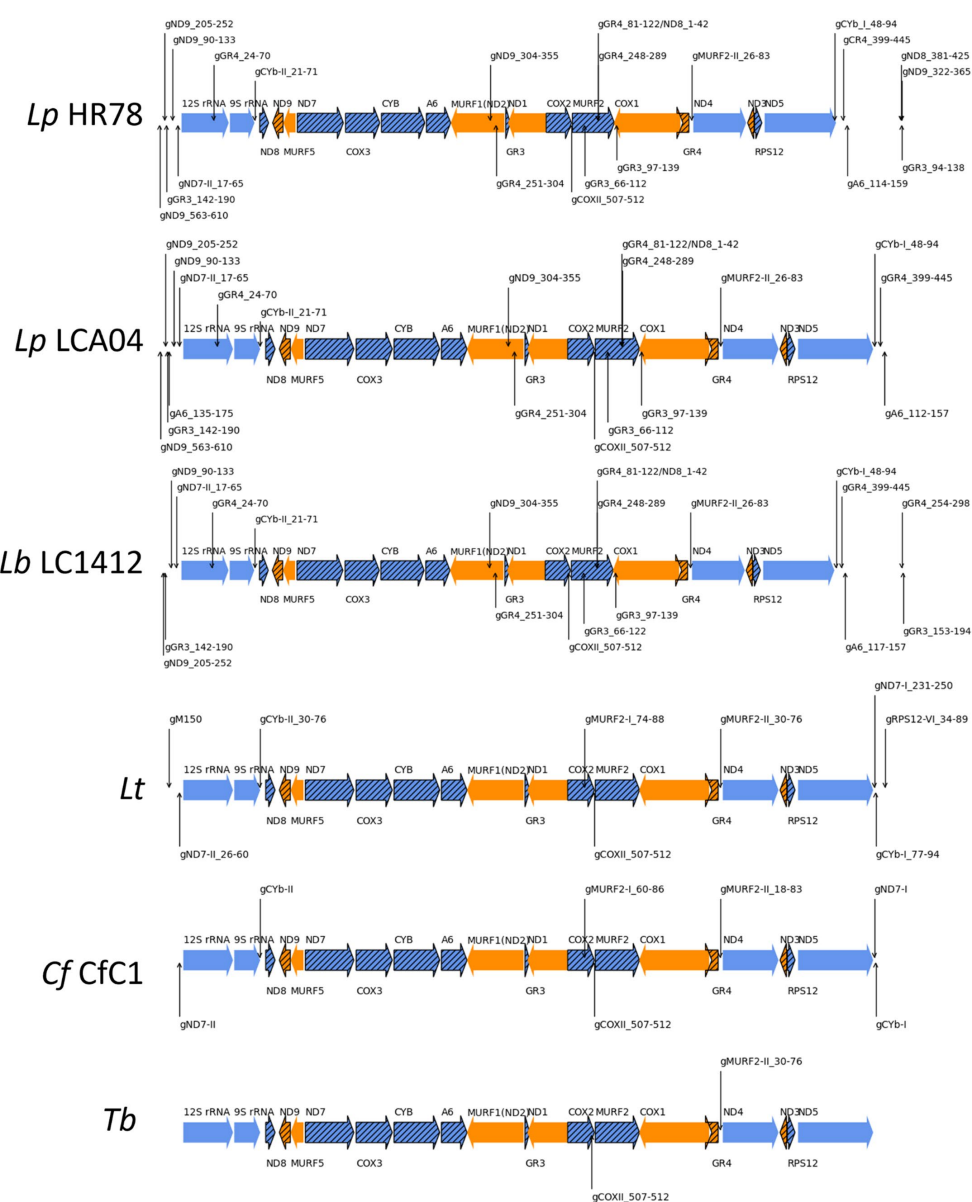
Comparison of maxicircle‐encoded gRNAs in trypanosomatids. The synteny of all rRNA and protein‐coding genes and of some gRNA genes is conserved. The gRNAs are labeled with Roman numerals or nt position on edited mRNA, based on the study in which they are first described. Abbreviations and references: Tb: *T. brucei* EATRO1125 (Cooper et al. 2019), Cf: *C. fasciculata* CfC1 (Van der Spek et al. 1991), Lt: *L. tarentolae* (Blum et al. 1990), Lp: *L. peruviana* HR78 and LCA04 (Van den Broeck et al. 2020), Lb: *L. braziliensis* LC1412 (Van den Broeck et al. 2020).

It is important to note that gRNA genes are also encoded within maxicircle molecules. In fact, the first gRNA genes were identified within the *L. tarentolae* maxicircle (Blum et al. 1990). A comparison of maxicircle‐encoded gRNAs between trypanosomatids reveals interesting differences (Figure 9). In *T. brucei* , only two gRNA genes are found on the maxicircle, including the gRNA encoded *in cis* within the COX2 mRNA 3′ UTR (Golden and Hajduk 2005). So far, nine maxicircle‐encoded gRNAs have been found in *L. tarentolae*, eight directing U‐indels on CYb, COX2, MURF2, ND7, and RPS12, and one remaining unassigned (Blum et al. 1990; Maslov and Simpson 1992). Seven maxicircle‐encoded gRNA genes with conserved synteny to those in *L. tarentolae* have been described in *C. fasciculata* (Van der Spek et al. 1991). A much greater number of 19 to 21 putative gRNA genes were identified within the maxicircles of *L. peruviana* and *L. braziliensis* , often located near or within the mito‐ribosomal rRNA genes or just downstream of the conserved protein coding region (Van den Broeck et al. 2020). Thus, the number of maxicircle‐encoded gRNA genes appears to increase in later‐branching trypanosomatids, and expanding such a comparison to a wider range of kinetoplastid species (Kostygov et al. 2023) promises to provide important clues about the emergence and evolution of gRNA genes.

### 6.3 Guide RNA Repertoires

Near‐complete repertoires of gRNA loci‐containing molecules were critical to the elucidation of repertoires of actual gRNAs. Another requirement was the ability to reconstruct all mitochondrial edited mRNA open reading frames in a species of interest. This is challenging due to the presence, in high throughput sequencing reads, of non‐canonical editing patterns inconsistent with those found in translatable, edited products, but is now possible with the *T‐Aligner* software package (David et al. 2015; Gerasimov et al. 2021, 2018). The package was further developed to align all edited mRNA sequences with all gRNA‐containing molecules within a single strain or species. Regions of minicircles (or their equivalent) aligning with edited mRNAs, either predicted through tools such as *T‐aligner* or using sets of canonically edited sequences, represent the putative gRNA loci. Once these were identified, nearby sequence motifs can be definitively identified and used as a discriminator for likely‐ versus pseudo‐gRNA loci. Sequencing of small mitochondrial RNA reads validate these gRNA loci, typically with impressive agreement (Afonin et al. 2024; Cooper et al. 2019, 2022; Gerasimov et al. 2021) and provide evidence of expression. These approaches reveal that gRNAs characteristics of length, frequency of G:U pairing, number of allowable mismatches to final edited products, and anchor (initial binding) region requirements clearly differ between species (Table 2). For instance, *L. pyrrhocoris* gRNAs are shorter with higher mismatch tolerance than *L. tarentolae* gRNAs (Gerasimov et al. 2021). It is therefore possible that as‐of‐yet undetected minor species differences in binding capabilities of RESC have co‐evolved with differences in gRNA characteristics. Small mitochondrial transcriptome analysis of *T. brucei* has facilitated a quantitative description of the features of gRNA gene cassettes and gRNA genes themselves, including mean lengths for the AT‐rich gRNA initiation sequence, anchor, and guiding region (Figure 8, top) (Cooper et al. 2022). This analysis revealed that the combined length of initiation sequence and anchor is remarkably conserved at 15–19 nt. Combined with the crucial recognition of the gRNA 5′ triphosphate by RESC, this conserved feature could serve as an important ‘molecular ruler’ in the selection of functional gRNAs for RNA editing initiation (Liu et al. 2023). Mapping identified gRNA loci back to fully edited transcripts has also revealed high species divergence in functional redundancy of gRNA repertoires. It is highest in *T. borreli* (where on average over 8 gRNAs have the potential to direct a specific insertion or deletion event) (Gerasimov, Afonin, et al. 2022) and in pleomorphic strains of *T. b. brucei* (Cooper et al. 2019), and lowest in *B. nonstop* and *T. brucei gambiense* type I with virtually no redundancy (Afonin et al. 2024; Geerts et al. 2022). *B. nonstop* also has the smallest number of identified gRNA loci, which explains its low redundancy. However, this relationship in general is not absolute, as factors such as the number and extent of transcripts requiring editing also affect redundancy. Guide RNA mapping also reveals “cryptic” gRNA loci, encoding gRNAs not obviously capable of directing editing of any productively edited transcript, yet often with proof of transcription (Afonin et al. 2024; Cooper et al. 2022; Simpson et al. 2015) (see also section 6.2). In at least some cases, these may be gRNAs previously utilized to direct editing of mRNAs that are no longer needed (Gerasimov et al. 2025) or gRNA genes that through mutations have drifted away from being functional or from meeting the cut‐off that was applied to call a match (Cooper et al. 2019).

### 6.4 Species‐Specific Features of RNA Editing

Concerning the overall characteristics of editing, some features are conserved, while others differ across species. Maxicircle transcript abundance is consistently highest for genes that are edited, likely so that adequate mature cryptogene mRNAs are generated despite inefficiency in editing (Gerasimov, Afonin, et al. 2022; Gerasimov, Ramirez‐Barrios, et al. 2022). However, the amount of “non‐canonical” insertions or deletions inconsistent with the pattern essential to an open reading frame is species‐specific. Non‐canonical editing events are most common in *T. borreli*, appearing in up to 95% of edited sequence reads, less abundant in *T. cruzi* , and occur even less in *L. pyrrhocoris* (Gerasimov, Afonin, et al. 2022). They are presumed to exist mainly within partially‐edited transcripts between the edited and yet‐to‐be edited regions, but this has yet to be confirmed in species outside of *T. brucei* . Most *L. pyrrhocoris* gRNAs were found to have the capacity to direct both canonical editing and editing of non‐canonical sequences appearing in the read population, even on an entirely different transcript (Gerasimov et al. 2021). As described in Section 4, developmental editing regulation in *T. brucei* involves the control of gRNAs for canonical editing and gRNAs that moonlight for non‐canonical editing, via mechanisms that require REH2C (Meehan et al. 2024, 2023). These variations in either stochastic or fixed use of non‐canonical editing could depend on the permissiveness or active regulation of gRNA:mRNA binding.

Another example of divergence among species is that while editing universally results in more additions than deletions, their ratios differ. To generate open reading frames, *T. borreli* mitochondrial mRNAs utilize on average 3.3 insertions for every deletion, while this ratio is 5.1 in *T. cruzi* and 9.1 in *L. pyrrhocoris* (Gerasimov, Afonin, et al. 2022). This editing quality could most logically be traced to the editing factors themselves, such as species‐specific differences in RECC terminal uridyl transferase and ribonuclease activity. It will take additional sequencing efforts to elucidate possible relationships between these characteristics of editing quality, gRNA characteristics, organism lifestyle, and taxonomic relationships.

Finally, deep sequencing analysis of U‐indel editing also reveals differences in gRNA‐linked loss of productive editing. Despite the apparent dispensability of electron chain complex I for many culture‐adapted or natural kinetoplastid lifestyles (Gerasimov et al. 2025; Surve et al. 2017; Simpson et al. 2015; Surve et al. 2012; Thiemann et al. 1994), mitochondrial genes and cryptogenes of complex I subunits often remain encoded and their transcripts expressed. *L. tarentolae* culture cultivated long term revealed the possibility that gRNA loss could drive complex I subunit mRNA editing loss, resulting in the loss of complex I (Thiemann et al. 1994). Deep sequencing has since shown species‐specific qualitative differences in how this occurs, but that specific gRNA losses always propel the process. For instance, the loss of *V. ingenoplastis* complex I subunit mRNA‐specific gRNAs is predictably paired with reduced percentages of edited reads associated with complex I subunit cryptogenes (Gerasimov et al. 2025). In contrast, despite complex I activity not being discernable, *B. nonstop* complex I cryptogene mRNAs are still highly edited—just not consistently leading to normal open reading frames (Afonin et al. 2024). The low numbers of *B. nonstop* total encoded gRNAs clearly reveal that complex I subunit associated gRNAs do not exist. However, for *B. nonstop*, shorter (and more likely transient) alignments of the cognate gRNAs of its other transcripts with the “random” editing patterns on complex I subunit transcripts show that they may be largely responsible for directing these “nonproductive” editing patterns. This phenomenon is even more intriguing given that noncanonical gRNAs only introduce random editing patterns in cryptogene transcripts, apparently avoiding correctly encoded transcripts as targets. Thus, there are still factors governing the associations of editing factors with mRNA templates and gRNAs that we have yet to understand. While we have illustrated that differences in gRNA use in response to changing nutrient access are apparent within the two host (dixenous) species, the continued analysis of editing across kinetoplastids will make the story more complete.

## 7 Evolution of RNA Editing in Kinetoplastids and Diplonemids

**FIGURE 10:**
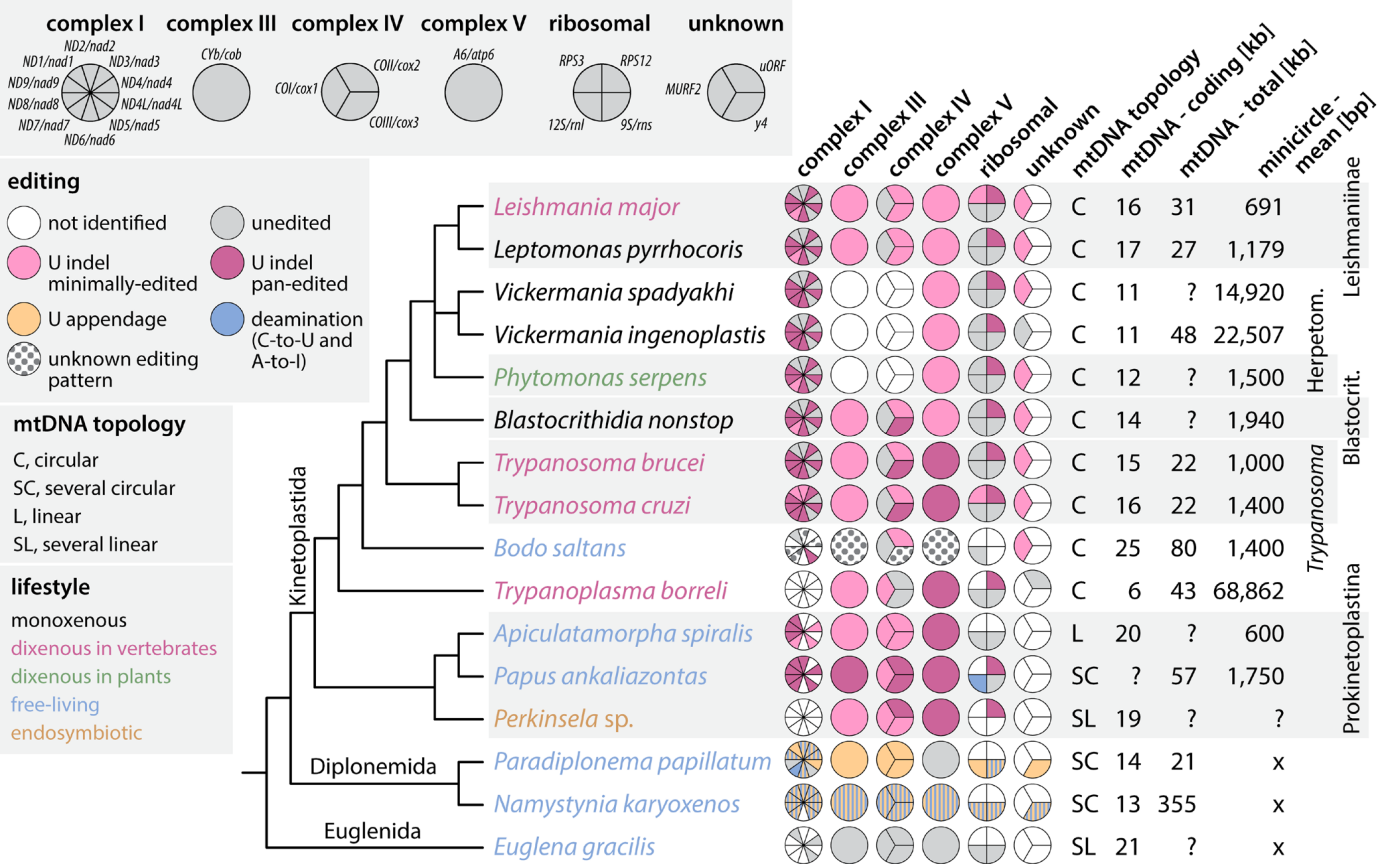
A representative overview of mitochondrial DNA organization, RNA editing patterns, and lifestyles in Euglenozoa. Genes are named as established in kinetoplastids and/or diplonemids. Note that the *MURF2* and *uORF* genes are found only in kinetoplastids, whereas the *y4* gene is confined to diplonemids. Abbreviations: Blascrith., Blastocrithidiinae; Herpetom., Herpetomonadinae; mtDNA, mitochondrial DNA; U indel, U insertion/deletion;?, unknown; x, not present.

To understand how the unique, extremely complex machinery needed for mitochondrial RNA editing in trypanosomes and other Kinetoplastea evolved, we might start with a comparative analysis of their closest relatives, the sister clades Diplonemea and Euglenida, with the latter branching off first (Kostygov et al. 2021). All these flagellate protists belong to the supergroup Discoba, now considered one of the two most basal branches of the eukaryotic tree (Williamson et al. 2025). In the euglenid model species *Euglena gracilis* , mitochondrial RNA does not undergo U‐indel editing (Figure 10), ruling out speculations regarding this process as a remnant of an RNA world (Gilbert 1986). However, 14 putative homologs of subunits of various editosomal complexes functionally characterized in *T. brucei* have been found in the mitoproteome of *E. gracilis* , though their functions are still unknown. Two pentatricopeptide (PPR) proteins, six RNA helicases, and a handful of other proteins possibly associated with mitochondrial RNA are present as well (Hammond et al. 2020). These proteins and rampant scrambling of both mitochondrial genome and transcriptome (Flegontov et al. 2011) might have lain at the ancestral basis of ever‐increasing complexification.

Such further complexification can be observed in diplonemids, for instance in their genetically tractable representative *Paradiplonema* (*Diplonema*) *papillatum*, for which the whole genome and transcriptome are available (Valach, Moreira, et al. 2023), and several other diplonemid species (Yabuki et al. 2016). Their mitochondrial RNA is subject to the most complex combination of post‐transcriptional processing known, composed of extensive *trans*‐splicing, as well as appendage and substitution editing (Kaur et al. 2020; Yabuki et al. 2016). Unexpectedly, mitochondrial genome structure and RNA processing strongly deviated from the kDNA structures and U insertion/deletion editing found in kinetoplastids (Valach et al. 2014), and kinetoplastid‐like gRNAs have not been found, despite intensive searches (Moreira et al. 2016). As observed in euglenozoans, diplonemid mitoproteomes also contain proteins resembling editosomal components, like two terminal uridylyl transferases (TUTases) (Gray et al. 2025), one with a predicted 3D structure very similar to RET1 and RET2 (Rajappa‐Titu et al. 2016). Most surprisingly, the *P. papillatum* mitoproteome also contains more than a hundred PPR proteins (Gray et al. 2025), a number comparable with the one found in plant mitochondria and chloroplasts (Small et al. 2020), evolving independently. Though experimental evidence is still lacking, these PPR proteins, with numbers in the range of kinetoplastid gRNA complements, are suspected to be involved in assemblage and specific deaminations of fragmented mitochondrial transcripts (Gray et al. 2025).

Both diplonemids and kinetoplastids evolved giant mitochondrial genomes, resembling other eukaryotic “junk” DNA, measuring hundreds of Mbp, but with coding potentials of organellar genomes about a thousand times smaller (Lukeš et al. 2018). In both groups, the few protein‐coding genes are split into separately encoded segments or into the gene encoding an unedited transcript and its associated gRNA genes, respectively, needing *trans*‐splicing and/or extensive editing. Whether the bloated genome sizes are linked to the intricate, complex, post‐transcriptional processing remains an open question.

Mitoribosomes and mitochondrial‐encoded subunits of the respiratory complexes differ between diplonemids and kinetoplastids, but still maintain marks of shared evolutionary history (Ramrath et al. 2018; Valach, Benz, et al. 2023). However, differences between these sister groups' DNA polymerases and other proteins involved in the maintenance and replication of mitochondrial DNAs are much more pronounced. Could it be that the distinct evolutionary paths since their split (a few hundred million years ago) taken by the diplonemid and kinetoplastid mitochondrial genomes, transcriptomes and post‐transcriptional processing tell us something about what shaped the kinetoplastid editing machinery?

**FIGURE 11:**
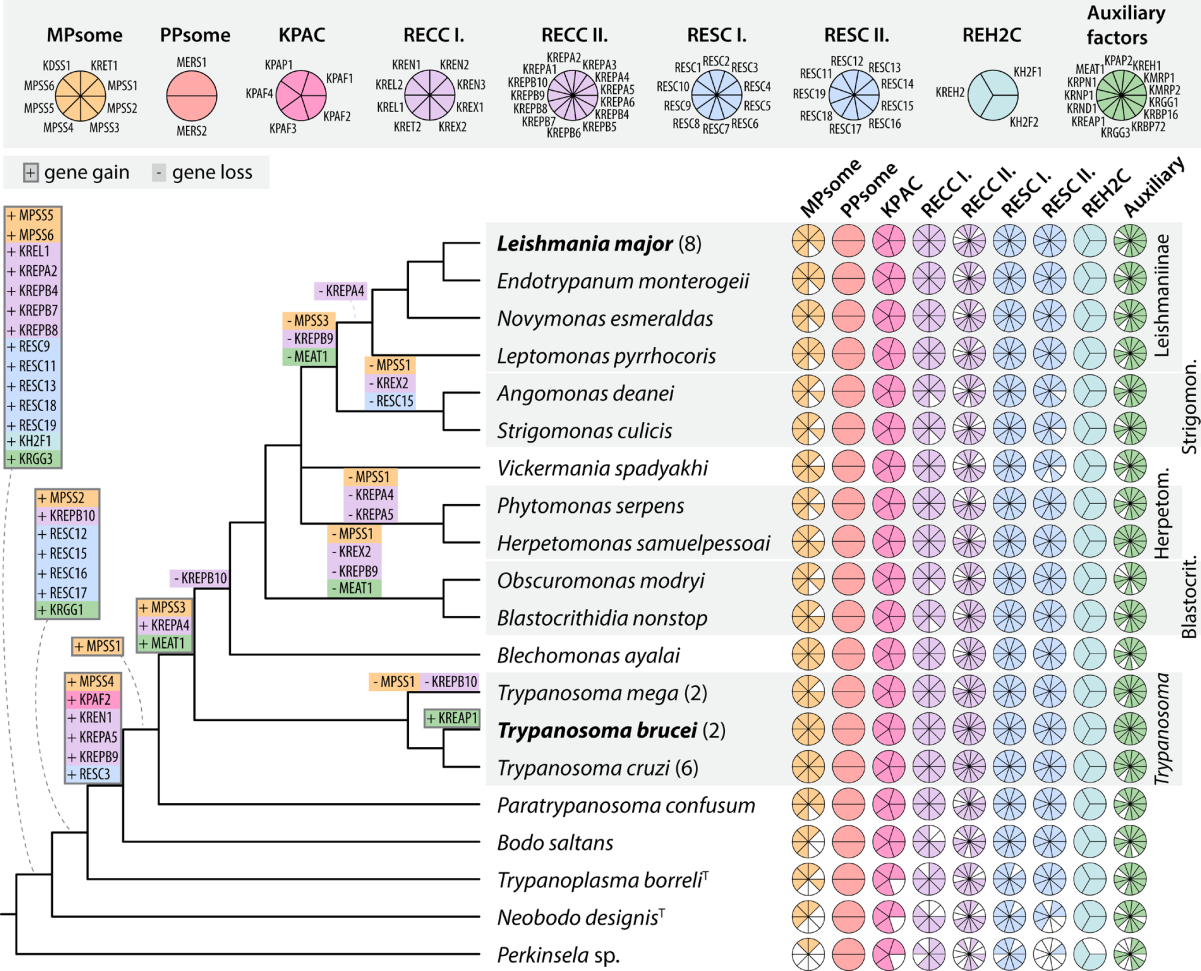
An overview of the distribution of protein complexes and auxiliary factors involved in RNA editing in Kinetoplastea. For visualization purposes, RECC and RESC complexes were split into two Coulson plots. *L. major* and *T. brucei* are in bold as kinetoplastid model species. Numbers of species with the same composition of editosome (for *L. major* , all four subgenera are included; for *T. mega*, also *T*. (*Haematomonas*) *boissoni* is included; for *T. brucei* , also *T*. (*Duttonella*) *vivax* is included; for *T. cruzi* , also *T*. (*Squamatrypanum*) *platydactyli, T*. (*Crocotrypanum*) *grayi, T*. (*Megatrypanum*) *theileri*, and *T*. (*Trypanomorpha*) *avium* are included) are in parenthesis. Abbreviations: Blastocrit., Blastocrithidiinae; Herpetom., Herpetomonadinae; KPAC—kinetoplast polyadenylation complex; MPsome—mitochondrial processome; PPsome—pyrophosphohydrolase complex; RECC—RNA editing core complex; RESC—RNA editing substrate binding complex; REH2C—RNA editing helicase 2‐associated complex; Strigomon., Strigomonadinae.

Though its absence from diplonemids and euglenids almost certainly rules out the emergence of U‐indel RNA editing prior to the separation of the kinetoplastid lineage, it does seem to have emerged before the acquisition of parasitic lifestyles (see Figure 10). Indeed, the early‐branching bodonids carry gRNAs (Blom et al. 2000) and subunits of all editosomal protein complexes (David et al. 2015). Looking at the most basal kinetoplastid studied, *Perkinsela* sp. (Figures 10 and 11), which branched off over one billion years ago (Lukeš et al. 2014), we can tentatively conclude that some form of post‐transcriptional RNA editing is old, and was already functional at that time, though probably not as extensive as nowadays (see below). This endosymbiotic kinetoplastid and the related ectoparasite *Ichthyobodo* contain enormous amounts of structurally disordered kDNA (~248 Mbp in *Perkinsela*) in their single inflated mitochondrion (Lukeš et al. 2018). Disk‐shaped kDNA, similar to present‐day kDNA in trypanosomes, has appeared, based on paleontological evidence, at least ~110 million years ago (Poinar and Poinar 2004). However, a sophisticated editing machinery almost certainly predates the arrangement of kDNA into a densely packed network (compare Figure 10).

The presence of all editosomal protein complexes in every kinetoplastid studied so far (Figure 11) can be explained by different scenarios: lineages with early, intermediate stages of the process went extinct, for example, because of the rapidly increasing complexity of this ratchet‐like process (see below). Our coverage of kinetoplastid diversity is still incomplete. However, we can safely conclude that basal and crown kinetoplastids have very similar U‐indel editing machineries, differing only somewhat in the number of identified subunits in the individual complexes (Figure 11). The difference is exemplified by a comparison between the smallest and largest sets of such identified components in *Perkinsela* and *T. brucei* , respectively (Figure 11). Of note, the former species has the most reduced nuclear genome by far among kinetoplastids (Tanifuji et al. 2017) and is the only known eukaryote totally devoid of introns, with *cis*‐splicing being absent, though *trans*‐splicing remains (Kostygov et al. 2024).

This brings us to the most hotly debated aspect of kinetoplastid editing: how and why did this incredibly complex editing machinery and its extravagant number of associated gRNAs evolve? An important aspect has to be highlighted first. The reversal of extensive editing by reverse transcription of edited mRNA was originally proposed to occur (Landweber 1992). This made an explanation by a neutral ratchet‐like mechanism, such as constructive neutral evolution (CNE), on its own, less likely. However, the absence of any experimental proof for such reversals leaves CNE as the most plausible null hypothesis. Thus, the kinetoplastid flagellates may just have become completely dependent on an increasingly complex repair mechanism. Rapid increase makes sense, for example, because a larger repertoire of gRNAs already makes it more likely that new to‐be‐edited sites can arise in the absence of large fitness costs. Indeed, numerous such unassigned gRNAs, with extensive “reserve” annealing potential, have been documented in several trypanosomatid species (Gerasimov et al. 2025, 2018; Tylec et al. 2019).

Such a rapid one‐way street aspect has surprising consequences. When looking at the editing patterns of Figure 10, rather than focusing on what organisms seem to have in common as being derived from an ancestor, we should instead watch out for instances of “absence of editing” to reconstruct the ancestral state. For example, because all three mitochondrial‐encoded subunits of respiratory complex IV occur in unedited form, RNA editing presumably did not start out in any of their transcripts.

Though the origins of U‐indel editing may, in the absence of intermediate stages in the extant organisms, remain intractable, the proposed repurposing of pre‐existing activities, such as TUTases, endonucleases, and exo‐U‐ases (Covello and Gray 1993), in the ancestor of all euglenozoans seems likely. Yet such repurposing has taken dramatically different paths. The radically divergent systems that evolved in diplonemids, seemingly dependent on hundreds of distinct PPR proteins, and kinetoplastids, relying on hundreds of gRNAs, are subject to different evolutionary forces, but still ended up with highly complex examples of CNE. What may have triggered it? Glycosomes, somewhat repurposed incarnations of peroxisomes, are shared by kinetoplastids and diplonemids. They have evolved differently (Hammond et al. 2025), but both seem indicative of rapid changes in mitochondrial activity when confronted by fluctuations in carbon sources and/or oxygen availability (e.g., by allowing rapid autophagy of the protein‐rich, metabolically specialized organelle) (Cull et al. 2014). In combination with eukaryotic population bottlenecks, temporary mitochondrial inactivity might have been the perfect breeding ground for the start of major deviations such as the RNA editing process (Cavalier‐Smith 1997; Speijer 2023).

It seems useful to delve a little bit further into CNE. It has been observed before that trypanosomes so often perfectly illuminate biological processes (Lukeš et al. 2023), and this holds true in this instance too. Nobody has problems accepting chance in the form of random mutations as the basis selection can work on. On macro levels, as with the vertebrate inverted retina, we also accept the surprising effects of chance. However, with fundamental cellular processes we find layers of “useless” complexity hard to acknowledge. Kinetoplastid RNA editing demonstrates the power of chance at this level as well. This does not mean that selection does not enter the picture: “repair” by editing must, in individual cases, not have large fitness costs, though it might cumulatively influence the available options. Might this explain the overall tendency toward parasitism in this group? Although there are no obvious links between the extent of editing and lifestyle (Figure 10), the complex life cycle of, and population bottlenecks encountered by, trypanosomes and leishmanias that alternate between invertebrate and vertebrate hosts may cause increased incidence of U insertions and/or deletions. In order to cope with that, an increasingly complex machinery might be needed as well (Figure 11). Thus, despite alternative “selectionist” explanations (Speijer 2008), rampant CNE seems to fit the available data best (Lukeš et al. 2011).

## 8 Conclusions and Future Perspectives

Here, we comprehensively synthesize current knowledge and perspectives for the functions, developmental regulation, and evolution of mitochondrial RNA editing protein complexes and their mRNA and gRNA substrates in kinetoplastids. There are several key outstanding questions. First, while RECC, RESC, and REH2C, and several auxiliary protein components have been catalogued and functionally annotated (Aphasizheva et al. 2020; Gazestani et al. 2016) (Table 1), there are likely numerous unidentified proteins that directly participate in editing or its regulation. The definition of an editing factor can be extended, since proteins such as the RNA helicases KREH2 and KH2F1 are not only required for general editing function but also mediate state‐ and substrate‐specific repression of editing. Additional proteins, including those that incorporate RNA modifications, may be required to regulate‐specific transcripts (McDermott et al. 2023; Sprehe et al. 2010) or even editing sites. Second, while RECCs, RESCs, and REH2Cs mediate catalysis, substrate organization, and developmental regulation, respectively, the precise molecular mechanisms of site‐ and substrate‐specific recognition, as well as the step‐by‐step assembly of complexes at various stages of the process, remain to be determined. The exact molecular function of most domains in these editing complexes remains unknown, and proteins lacking recognizable motifs are especially difficult to study. In addition, the detailed kinetics and dynamics of interactions within and between these editing complexes and their mRNA and gRNA substrates are still obscure, as are those with the factors that are required for up‐ and downstream RNA processing and translation, for example, with diverse pentatricopeptide repeat (PPR) proteins that control 5′ and 3′ modifications of mitochondrial mRNAs (Aphasizheva et al. 2020, 2011, 2016; Etheridge et al. 2008; Kao and Read 2005; Mesitov et al. 2019; Zhang et al. 2017). Third, how precisely REH2C differentially controls the utilization of gRNAs in canonical editing and novel gRNAs that moonlight to achieve stage‐ and substrate‐specific termination of editing remains unknown. Fourth, how exactly RECCs recognize U‐deletion and U‐insertion sites, and how RECC isoforms switch between each other, and possibly control site‐ and substrate‐specificity, remains unclear. Importantly, site‐ and substrate‐specificity, as well as their regulation, must also be coordinated with other large‐scale changes in mitochondrial biology and nuclear gene activity (Walsh and Hill 2021; Zíková 2022). We expect that future analyses of mitochondrial genomes, editing, and editing complexes across kinetoplastids in general and trypanosomatids in particular, including those with distinct life cycle stages and evolutionary histories, will reveal crucial insights into editing regulation during development, including how they are integrated more broadly with environmental change and cellular differentiation. We anticipate that the recent advances in AI modeling for multi‐omic data integration and protein interaction prediction will be instrumental in answering all the above questions and more.

## Author Contributions

**Suzanne M. McDermott:** conceptualization (equal), visualization (equal), writing – original draft (lead), writing – review and editing (lead). **Julius Lukeš:** conceptualization (equal), visualization (equal), writing – original draft (equal), writing – review and editing (equal). **Laurie K. Read:** conceptualization (equal), visualization (equal), writing – original draft (equal), writing – review and editing (equal). **Reza Salavati:** conceptualization (equal), visualization (equal), writing – original draft (equal), writing – review and editing (equal). **Achim Schnaufer:** conceptualization (equal), visualization (equal), writing – original draft (equal), writing – review and editing (equal). **Sara L. Zimmer:** conceptualization (equal), visualization (equal), writing – original draft (equal), writing – review and editing (equal). **Jason Carnes:** conceptualization (supporting), visualization (supporting), writing – original draft (supporting), writing – review and editing (supporting). **Alasdair Ivens:** conceptualization (supporting), visualization (supporting), writing – original draft (supporting), writing – review and editing (supporting). **Naghmeh Poorinmohammad:** conceptualization (supporting), visualization (supporting), writing – original draft (supporting), writing – review and editing (supporting). **Nicholas J. Savill:** conceptualization (supporting), visualization (supporting), writing – original draft (supporting), writing – review and editing (supporting). **Dave Speijer:** conceptualization (supporting), visualization (supporting), writing – original draft (supporting), writing – review and editing (supporting). **Ken Stuart:** conceptualization (supporting), visualization (supporting), writing – original draft (supporting), writing – review and editing (supporting). **Kristína Záhonová:** conceptualization (supporting), visualization (supporting), writing – original draft (supporting), writing – review and editing (supporting). **Poorya Mirzavand Borujeni:** conceptualization (supporting), visualization (supporting), writing – original draft (supporting), writing – review and editing (supporting). **Zihao Chen:** conceptualization (supporting), visualization (supporting), writing – original draft (supporting), writing – review and editing (supporting). **Cody Goode:** conceptualization (supporting), visualization (equal), writing – original draft (supporting), writing – review and editing (supporting). **Sunil Kumar Sharma:** conceptualization (supporting), visualization (supporting), writing – review and editing (supporting). **Lars O’Hara:** conceptualization (supporting), visualization (supporting). **Jorge Cruz‐Reyes:** conceptualization (lead), visualization (lead), writing – original draft (lead), writing – review and editing (lead).

## Funding

This work was supported by National Science Foundation 1616845 to J.C.R.; National Science Foundation 2140153 to S.M.; National Institutes of Health R01 AI184920 to J.C.R.; National Institutes of Health R21 AI187936 to J.C.R.; National Institutes of Health R01 GM129041 to L.K.R.; Natural Sciences and Engineering Research Council of Canada RGPIN‐05904 to R.S.; Canadian Institutes of Health Research 252733 to R.S.; National Institutes of Health R01 AI014102 to K.S, and the Czech Grant Agency 25‐15298S to J.L. and K.Z.

## Conflicts of Interest

The authors declare no conflicts of interest.

## Related WIREs Articles

Trypanosome RNA editing: the complexity of getting U in and taking U out.

Dynamic RNA holo‐editosomes with subcomplex variants: Insights into the control of trypanosome editing.

## Data Availability

Data sharing is not applicable to this article as no new data were created or analyzed in this study.
